# Fully Floatable Mortise‐and‐Tenon Architecture for Synergistically Photo/Sono‐Driven Evaporation Desalination and Plastic‐Enabled Value‐Added Co‐Conversion of H_2_O and CO_2_

**DOI:** 10.1002/advs.202404423

**Published:** 2024-05-20

**Authors:** Yingying Li, Tongrong Yao, Yanqiu Wang, Jiahui Chen, Haining You, Jing Lu, Yi Xiong, Zhongduo Xiong, Jia Liu, Yajuan Qi, Wenwen Wang, Dong Wang

**Affiliations:** ^1^ Key Laboratory of Textile Fiber and Products Ministry of Education Wuhan Textile University Wuhan 430200 China; ^2^ Multifunctional Electronic Ceramics Laboratory College of Engineering Xi'an International University Xi'an 710077 China; ^3^ College of Science Wuhan University of Science and Technology Wuhan 430081 China

**Keywords:** H_2_O and CO_2_ reduction, mortise‐and‐tenon joint structure, photo/sono catalytic plastics degradation, plastics upcycling, water evaporation

## Abstract

Establishing an advanced ecosystem incorporating freshwater harvesting, plastic utilization, and clean fuel acquisition is profoundly significant. However, low‐efficiency evaporation, single energy utilization, and catalyst leakage severely hinder sustainable development. Herein, a nanofiber‐based mortise‐and‐tenon structural Janus aerogel (MTSJA) is strategically designed in the first attempt and supports Z‐scheme catalysts. By harnessing of the upper hydrophilic layer with hydrophilic channels embedding into the hydrophobic bottom layer to achieve tailoring bottom wettability states. MTSJA is capable of a fully‐floating function for lower heat loss, water supply, and high‐efficiency solar‐to‐vapor conversion. Benefiting from the ultrasonic cavitation effect and high sensitivity of materials to mechanical forces, this is also the first demonstration of synergistic solar and ultrasound fields to power simultaneous evaporation desalination and waste plastics as reusable substrates generating fuel energy. The system enables persistent desalination with an exceptional evaporation rate of 3.1 kg m^−2^ h^−1^ and 82.3% efficiency (21 wt.% NaCl solution and 1 sun), and realizes H_2_, CO, and CH_4_ yields with 16.1, 9.5, and 3 µmol h^−1^ g^−1^, respectively. This strategy holds great potential for desalination and plastics value‐added transformation toward clean energy and carbon neutrality.

## Graphical abstract

This is the first demonstration of strategically designed fully floating mortise‐and‐tenon structure Janus aerogels to support Z‐type heterojunction, and also for the first time, utilizing the incorporation of synergetic photo and ultrasound fields can achieve evaporation desalination and catalytic plastic as feedstocks for the value‐added transformation of H_2_O and CO_2_.

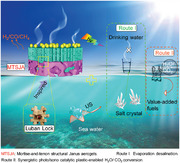

## 1 Introduction

In the not‐so‐distant future, freshwater will become pressing bulk luxuries due to irresponsible activities of white pollution and nuclear‐contaminated wastewater discharge et al.^[^ ^1^ ^]^ In multiple feasible strategies dedicated to freshwater acquisition,^[^ ^2^ ^]^ solar‐powered evaporation and photocatalytic decontamination, stored photos into chemical bonds, emerge tremendous potential because of low‐cost and clean‐sustainable capabilities.^[^ ^3^ ^]^ For solar evaporation, an advanced evaporator is rationally designed with skeleton structures serving as water transporting and photothermal materials functioning on thermal management, which are of particular importance components for high‐efficiency evaporation.^[^ ^4^ ^]^ Typical Janus‐structural evaporators as admired alternatives result from opposite wettability states,^[^ ^5^ ^]^ notwithstanding, these remain numerous challenges: inevitable heat loss due to hydrophilic layer immersion in water and salt deposition existence after long‐term utilization. The “mortise‐and‐tenon” structure, derived from ancient China, is considered to be inherited and promoted by the famous craftsman Master Lu Ban as one of the representatives in the Chinese architecture field.^[^ ^6^ ^]^ Two woodworking components ingeniously interlock between convex components (mortise) and concave parts (tenon), and the mortise matched insert into the hole of the tenon. Rarely are the mortise‐and‐tenon structure evaporators reported, nevertheless. An inspired evaporator, is strategically equipped with a hydrophilic top joint with a hydrophobic bottom, tailoring synergistic wettability bottom states by hydrophilic channels implanting into the hydrophobic bottom. Different from traditional Janus structures,^[^ ^7^ ^]^ the mortise‐and‐tenon structural device exhibits the following advantages: 1) conventional 3D water contact evaporation evolving into 2D water contact evaporation; 2) minimizing heat loss based on the fully floating function; 3) efficient water supply and photothermal conversion; 4) supporting catalysts for high stability and recycling.

For photocatalytic decontamination, plastic pollutants undoubtedly exacerbate the water resource issue.^[^ ^8^ ^]^ Waste plastic takes several centuries for natural degradation,^[^ ^9^ ^]^ and mostly enters the oceans forming microplastics that undergo bioaccumulation and biomagnification in the human body by the food chain and drinking water.^[^ ^10^ ^]^ Actually, plastic is a severely underestimated resource and could accomplish reclaimable utilization into valued products.^[^ ^11^ ^]^ For instance, poly(ethylene terephthalate) (PET) acts as an electron donor once hydrolyzed in the photocatalysis system to reduce the recombination of electrons and holes.^[^ ^12^ ^]^ Worth noting is that one prevalent approach to enhance H_2_O or CO_2_ conversion is constantly adding fresh sacrificial agents along with increasing costs.^[^ ^13^ ^]^ Given this, the co‐conversion of plastic as feedstock coupled with H_2_O and CO_2_ is a favored strategy to achieve carbon neutrality.^[^ ^12^, ^14^ ^]^ Uekert et al. obtained H_2_ fuel by photoreforming waste bottles;^[^ ^13a^ ^]^ Wang et al. promoted CO_2_ reduction from PET by green electrochemistry technology.^[^ ^13b^ ^]^ Unfortunately, it necessitates further breakthroughs of heavy reliance on single solar energy, limitations on transparency of wastewater, and inefficient mass transfer, causing lower photocatalytic activity.

Ultrasound, a periodic oscillatory mechanical wave emitted by marine organisms, is a common marine resource, extensively developed in sonodynamic therapy.^[^ ^15^ ^]^ Inspired by that, adopting ultrasound field optimizing solar energy technology opens up a novel idea to address some issues, which is fractionally involved. First, for solar‐to‐vapor extraction, it holds the potential to alleviate predicaments of salt accumulation. There is a lack of strong interface adhesion between salt crystals and evaporators, and it can accelerate the peeling and dissolution of salt particles by ultrasound resonance, facilitating long‐term seawater evaporation. Afterward, as for photocatalysis, it can be reinforced by ultrasound assistance based on powerful penetration and ultrasonic cavitation.^[^ ^16^ ^]^ Of note is that it develops cavitation bubbles emerging formation, expansion, and collapse, enabling the generation of “sonoluminescence” and “hot spots” with local high temperature‐high pressures to encourage forming free radicals.^[^ ^17^ ^]^ In addition, the buildup of piezoelectric potential results from the piezoelectric property of semiconductors in response to mechanical waves, rendering catalysts into an excited state.^[^ ^18^ ^]^ Consequently, the polarized electric field boosts sono‐generated radicals with powerful oxidation capability, such as hydroxyl radicals from water decomposition and superoxide radicals on the conduction band (CB) of semiconductors. Wang et al. reported a dual Z‐scheme structure with high carrier separation to enhance sonocatalytic levofloxacin removal.^[^ ^17a^ ^]^ Fang et al. demonstrated the porous structural HD‐CoNi‐LDH due to ultrasonic absorption and piezoelectric sono/photo catalytic H_2_ production.^[^ ^19^ ^]^ However, the high sound energy required is unfeasible for natural conditions, thus to exploit highly sensitive materials to the ultrasound field is necessary.

**Figure 1:**
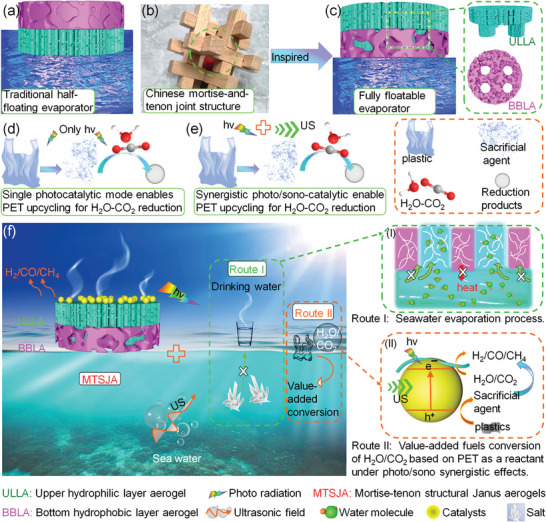
Schematic of the fully floatable MTSJA‐supported photo/sono catalysts nanocomposites. a) A traditional Janus evaporator consists of a bottom hydrophilic layer and upper hydrophobic structures. b) A protruding mortise joint with a concave tenon with interlock to offer a connecting function, in ancient Chinese Lu Ban lock architecture. c) A novel Janus evaporator inspired by mortise‐and‐tenon structure with upper hydrophilic and bottom hydrophobic layers. d) Plastic acts as feedstock for H_2_O or CO_2_ reduction, typically through photocatalysis. e) Synergistic photo/sono unique catalysis of plastic as a feedstock coupled with H_2_O‐CO_2_ conversion. f) Schematic of a typical fully floatable water evaporation system and catalytic fuel acquisition strategy: I) under photo/sono irradiation, a continuous vapor generation by harnessing photothermal conversion, water transport upward, and mechanical wave resonance for desalination; II) ultrasound‐assisted photocatalytic PET degradation as a sacrificial agent of an electron donor undergoing oxidation reaction to further propel the transformation of H_2_O and CO_2_ into fuel.

For the first time, a unique mortise‐tenon‐like inspired structural Janus aerogel (MTSJA) is fabricated with an upper hydrophilic layer embedding into a lower hydrophobic layer aerogel, which achieves photo/sono synergistically forcing evaporation desalination and PET reusable transformation for fuel acquisition (**Figure** **1**). The nanofiber‐constructed MTSJA with the upper layer built by nanofibers‐doped carbon nanotubes (CNTs), ensures photothermal conversion, beneficial water supply and minimizes heat loss from fully floating, and supports Z‐scheme heterojunctions of S─N co‐coped g‐C_3_N_4_ (SNCN) hybrid rGO quantum dots (GQDs) and CdS. Under photo/sono co‐irradiation, ultrasonic resonance can relieve salt accumulation, dramatically boosting evaporation desalination efficiency. Simultaneously, exploiting highly sound waves‐sensitive SNCN@GQDs/CdS heterojunctions and sono cavitation effect generates motivational impetuses, which improves photocatalytic reforming of PET into electron donors to facilitate fuel obtaining. Additionally, based on GQDs serving as an electron mediator, the Z‐scheme heterojunction constructed not only owns a stronger oxidation/reduction surface but also raises the separation and transfer of photo‐induced electron–hole pairs. There is no greater superiority than photo/sono synergetic evaporation desalination and PET conversion coupled with H_2_O‐CO_2_ reduction.

## 2 Results and Discussion

### 2.1 Construction and Characterization of the MTSJA Evaporator

**Figure 2:**
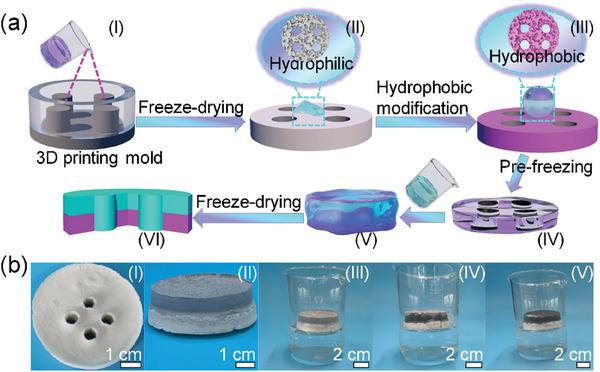
Overview of the MTSJA nanocomposites. a) Schematic of the fabricating process of the MTSJA aerogel, including the BBLA of concave tenon structure from (I) to (III) and ULLA of protruding mortise structure from (IV) to (VI). b) Photographs of BBLA (I), ULLA (II), and the self‐floatable MTSJA nanocomposites on the water surface.

The procedure of fabricating composite MTSJA is schematically illustrated in **Figure** **2** and Figure S1 (Supporting Information). The bottom hydrophobic layer aerogel (BBLA) with the self‐floating function with cellular‐shaped well structure is composed of commercial nanofibers (NFs) of polyvinyl alcohol and polyethylene (PVA‐*co*‐PE) by the utilization of a 3D printing mold (Figure 2b; Figure S2a,b, Supporting Information). The NFs go through a series of melt spinning, hydroxyl nucleophilic addition reactions with glutaraldehyde as a cross‐linking agent, freeze‐drying, and hydrophobic modification by the trichlorosilane to fabricate the BBLA (Figure 2a(I–III)). Based on its poor wettability performance, the BBLA is further immersed in isopropanol to pre‐freeze. Subsequently, the PVA‐*co*‐PE NFs bond with carboxyl‐functionalized CNTs by hydrogen bonds and cross‐link with glutaraldehyde to obtain a suspension, which are poured on the pre‐frozen BBLA with cellular‐shaped holes to construct the upper hydrophilic layer aerogel (ULLA) after a vertical freeze‐drying technology (Figure 2a(IV–VI)). ULLA with the hydrophilic channels filling into the hydrophobic honeycomb of BBLA achieves the MTSJA design (Figure 2b(I,II)). The unique MTSJA with the amalgamation of light absorbing from ULLA and adjusting wettability bottom capabilities from BBLA. It offers facile access for fully floating on the water surface, heat downward attenuation, and efficient water delivery, which is vitally important to break through traditional 3D water body contact to advance 2D water surface contact (Figure 2b(III–V)).

**Figure 3:**
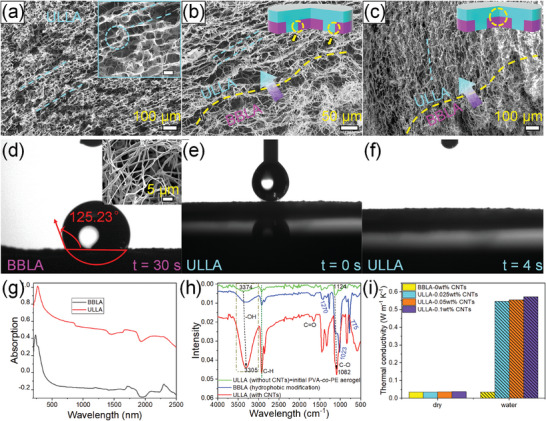
Characterization of the MTSJA. SEM images of the MTSJA showing ULLA with vertically aligned microchannel and the magnified vertical microchannel in the insert (a), and the interface morphology of BBLA‐ULLA (b,c). Photographs showing surface wetting behavior of BBLA (d) with the SEM image of unordered channels in the insert and ULLA (e,f), respectively. g) Absorption of the aerogel of BBLA and ULLA in UV–vis–NIR spectra. h) The FTIR molecular structure of representative BBLA, ULLA without CNTs (initial aerogel), and ULLA with CNTs. i) Thermal conductivity performance of ULLA and BBLA by controlling different contents of CNTs under dry and water conditions, respectively.

The SEM images of the freeze‐drying ULLA and the hydrophilic‐hydrophobic (ULLA‐BBLA) interface in the MTSJA system are displayed in **Figure** **3**a–c. Moreover, ULLA exhibits a 3D interconnected microporous structure, which consists of well‐designed vertically aligned continuous microchannels of 10–20 µm, and pore walls (1–2 µm) uniformly adhering CNTs on the NFs to compose an orderly striped wall structure. The porous walls are interconnected by porous bridges and nanofibers, and the micropore passages are formed between the walls. It implies capillary tubes formed to guide the upward transfer of water (Figure 3a). The network MTSJA aerogel with pores contributes to a high porosity of ≈96% and a low evident density of 13.8 kg m^−3^ (Figure S2c,d, Supporting Information). Additionally, interconnected boundary morphology and seamless interface between BBLA and ULLA are observed by the cross‐section characterization of the SEM images in Figure 3b,c. It suggests robust bonding and mutual penetration in the interface of hydrophilic‐hydrophobic layers. BBLA in the insert of Figure 3d has a 3D framework structure with micropore passages (10–20 µm) and pore‐like interconnected walls (0.5–2 µm), which is fabricated by PVA‐*co*‐PE NFs with sizes from dozens to hundreds of nanometers. By a series of dynamic contact angle measurements, the surface wettability behavior of every architecture involved in the preparation process of MTSJA is investigated, as exhibited in Figure 3d–f and Figures S3 and S4 (Supporting Information). In contrast to ULLA, the subsequent hydrophobically modified aerogel of BBLA exhibits a large contact angle with super hydrophobic performance, because of the occurrence of a large number of silane bonds to offer a rough surface and lower surface free energy (Figure 3d).

The light absorption properties in the solar spectrum of the MTSJA with different components are indicated in Figure 3g and Figure S5a (Supporting Information), illustrating notably stronger absorption of ULLA than that of BBLA and attributing to the superior absorption capacity over the broadband sunlight of CNTs in ULLA. Thermogravimetry is used to analyze the thermal capabilities of ULLA (Figure S5b, Supporting Information). To analyze the molecular structure and chemical constitutions, initial aldehyde cross‐linked PVA‐*co*‐PE aerogel (the green line), hydrophobically modifying PVA‐*co*‐PE ULLA (the blue line), and cross‐linked PVA‐*co*‐PE/CNTs hybrid ULLA (the red line) are characterized by FT‐IR (Figure 3h). All of the aerogels show matched typical characteristic peaks about PVA‐*co*‐PE at 2853–2930 cm^−1^ and 1332–1450 cm^−1^ relevant to the stretching and bending vibration of C─H. The characteristic peaks of the cross‐linked PVA‐*co*‐PE NFs sample located at ≈1541 and 1576 cm^−1^ are assigned to an acetal structure and stretching vibration peaks of alcohol ─OH at 3100–3500 cm^−1^. For the blue line, the difference is several functional group peaks emerged after undergoing hydrophobic modification, which illustrates some substituents of 775 cm^−1^ corresponding to Si─C bond stretching vibration, 1023 cm^−1^ attributing to Si─O─Si bond stretching vibration, and (Si)–CH_3_ symmetric bending vibration observing at 1270 cm^−1^. Moreover, it can be seen that some hydrophilic peaks decline and vanishing peaks, such as aldehydes. It reveals the forming hydrophobic interface in aerogel bonding through the cross‐linking points between the high reactivity of silicon–chlorine bonds in gaseous silane and hydroxyl bonds in NFs by covalent bonds. Furthermore, characteristic peaks in the red line exhibit consistent peaks with the green line, proposing the relatively few doped CNTs. The peaks of 3305, 1655, and 1082 cm^−1^ are stretching vibration of O─H, C═O, C─O. Compared with the initial PVA‐*co*‐PE aerogel, the characteristic peaks of the red line appear changes, especially the O─H and C─O groups own larger intensity and shift to short wavelength. It can prove that the aldehyde group, PVA‐*co*‐PE, and CNTs form binders through hydrogen bond interaction. The comparison of thermal conductivity shows the low thermal conductivity capability of BBLA, whether regarding water contact or in dry conditions, while it is opposite to ULLA with higher thermal conductivity in wet than in dry conditions (Figure 3i). It is not only superior to the conventional Janus hydrophilic bottom to restrict heat loss to the water body but also favored to temperature gradients between upper and lower layers, taking advantage of its porous 3D network structure, hydrophobic performance, and self‐floating characteristics.

### 2.2 Ultrasound‐Assisted Solar Vapor Generation Desalination

**Figure 4:**
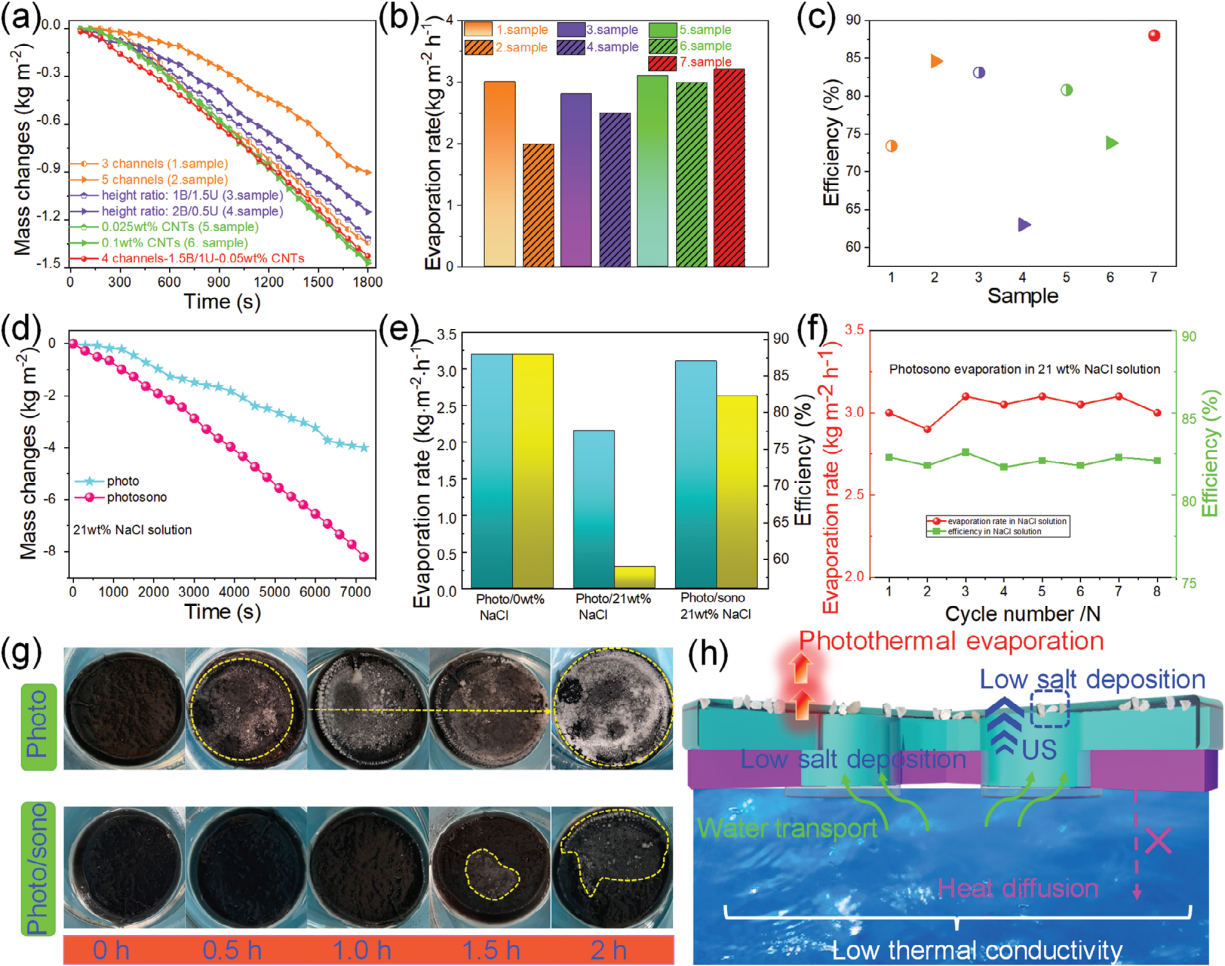
Performance of solar‐sono‐enabled evaporation desalination of MTSJA. a) Mass change evolution as a function of time of the MTSJA by regulating the numbers of hydrophilic channels, height ratio between BBLA and ULLA with a constant total height, and the contents of photothermal CNTs in the ULLA component under simulated 1‐sun irradiation. Calculated evaporation rates (b) and efficiency (c) of all the samples. d) Real‐time mass change in evaporation measurement of MTSJA under 21 wt.% NaCl solution by photo and photo/sono irradiation. e) Comparison of water evaporation rate and efficiency calculated under different conditions of photo/0 wt.% NaCl solution, photo/21 wt.% NaCl solution, and photo/sono/21 wt.% NaCl solution. f) Multiple cycle evaporation performance of MTSJA under photo/sono/21 wt.% NaCl solution. g) Photographs of salt precipitation process in the setup on the top surface of evaporators under 21 wt.% NaCl solution of photo and photo/sono irradiation. h) Schematical illustration of the ultrasound‐assisted solar‐enabled seawater desalination.

The performance of the solar vapor generation of the MTSJA is assessed. To investigate the thermal management ability of photothermal conversion and thermal insulation downward, the mass change and evaporation rate evolution are evaluated by controlling a series of parameters of the numbers of hydrophilic channels embedding into the BBLA, height ratio between BBLA and ULLA, and photothermal fillers of CNTs in ULLA (**Figure** **4**a,c). As can be seen, the numbers of hydrophilic channels implanted into BBLA are regulated to 3, 4, and 5, and the height difference between BBLA and ULLA are recorded as 1B/1.5U, 1.5B/1U, and 2B/0.5U. Besides, photo absorber CNTs are added with different doping amounts into ULLA. The time‐dependent mass evolution of water and surface temperature of aerogel are examined by electronic balance and infrared (IR) camera. The aerogel with 4 channels‐1.5B/1U‐0.05wt.% CNTs is considered as the optimal evaporation device to balance photothermal and thermal loss. To detect the temperature in real‐time, infrared images reveal an intuitive photothermal conversion phenomenon of MTSJA by adjusting the parameters (Figure S6, Supporting Information). The solar‐to‐steam conversion efficiency (*η*) of the MTSJA system can be calculated, as expressed by equations:^[^ ^20^ ^]^

(1) $$\eta \;=\;m\left(H_{\mathrm{LV}}+Q\right)/q_{1}C_{\mathrm{opt}}$$

(2) $$H_{\mathrm{LV}}=1.91846\times 10^{6}{\left[T/\left(T-33.91\right)\right]}^{2}\;\;J\;{\mathrm{kg}}^{-1}$$

(3) $$Q\;=\;c\left(T-T_{0}\right)$$

where *m* means the photo‐subtracting‐dark evaporation rate (kg m^−2^ h^−1^), *H_LV_* refers to the total latent enthalpy of the liquid‐to‐vapor phase transition of water (kJ kg^−1^), *c* represents the specific heat of water (4.2 J kg^−1^ K^−1^), *Q* signifies the sensible heat (J kg^−1^) within per unit temperature of the average surface temperature of system subtracting initial temperature of the water, and *q_1_* is related to the energy input of the incident light (kJ m^−2^ h^−1^). The results of the evaporation evaluation suggest that the aerogel is capable of achieving an excellent evaporation rate of 3.2 kg m^−2^ h^−1^ and energy conversion efficiencies of 88% calculated by tuning parameters (Figure 4b,c).

Normally, salt crystallization behavior continuously occurs and accumulates, attaching to hydrophilic walls and forming progressively denser structures during water transport and evaporation in real seawater, which seriously deteriorates the evaporation performance of fully floating evaporators accompanied by ultimately corroded nanostructures by salt. The ultrasound‐assisted photothermal evaporation behavior is examined to improve the salt‐resistant result with the mass loss performance by a factor of 2.2 for 2 h measurement compared with pure solar‐driven evaporation, in 21 wt.% NaCl solution under one sun irradiation (Figure 4d). It turns out that the improvement of photo/sono evaporation desalination is 44.2% and 39.7% superior to pure solar‐driven evaporation rate and efficiency in 21 wt.% NaCl solution, and slightly lower than pure solar‐powered 0 wt.% NaCl solution evaporation (Figure 4e). Thus, it shows relative durability and stability in the photo/sono‐powered evaporation desalination of cyclic tests (Figure 4f). To demonstrate the subtle thermal effects of ultrasound, the photothermal comparison images are demonstrated between photo and photo/sono evaporation desalination in 21 wt.% NaCl solution (Figure S7, Supporting Information). The corresponding surface temperature changes, cyclable photothermal functionality, and photo/sono‐driven evaporation desalination efficiency with adjustable solar and ultrasound powers are recorded (Figure S8, Supporting Information). The outdoor measurement of evaporation performance recorded up to 12 h is shown in Figures S9 and S10 (Supporting Information). Multiple metallic ions in seawater are compared with concentration changes by before and after desalination, which is far below drinking water standards (Figure S11, Supporting Information). It shows salt‐depositing action evolution on the surface of the evaporator in different photo and photo/sono evaporation approaches. More deposition of salt owing to the self‐stacking effect brings about severe salt corrosion after long‐term pure solar evaporation, which indicates salt tolerance of the photo/sono evaporator than the photo evaporator (Figure 4g). The ultrasound working time complied per 30 min on/off, which is much less than the light irradiation time. The efficient evaporation kinetics mechanism of high evaporation desalination performance can be summarized from the following aspects (Figure 4h). 1) The BBLA is endowed with low thermal conductivity and self‐floatable thermal barrier, effective water supply by the capillary channel effect functions. CNTs in the ULLA promote the spread of ultrasonic waves, and hydrophilic channels adsorb and retain water evaporation from salt solutions. 2) Ultrasonic waves increase interfacial activity and mass transfer rate between ultrasonic energy and salt crystals by the ultrasound cavitation effect of localized formation and collapse of tiny bubbles. 3) The matched frequency of ultrasonic waves and salt crystals evokes resonance, resulting in great energy input and energy propagation. 4) The high specific surface area and porosity structure of the MTSJA promote the diffusion rate of salt solution, transport paths, and numerous microchannels of dissolved salt. The lack of strong adhesion of the interface between MTSJA and salt crystals with vibration and fluffy accumulation accelerates the peeling and dissolution of the salt particles. The disappearance of salt is provoked by the salt‐redissolution competitively against the evaporation precipitation of brine to reach a dynamic equilibrium.

### 2.3 Photo/Sono Catalytic High‐Value Transformation of PET Coupled with H_2_O‐CO_2_

#### 2.3.1 Photo/Sono Catalytic PET Reaction

**Figure 5:**
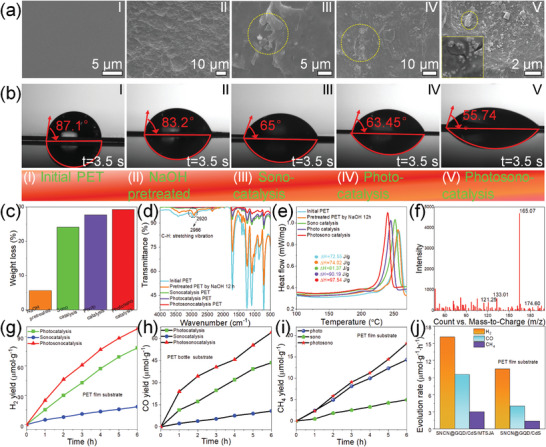
Synergistic photo/sono catalytic activity. SEM images a) and wettability at 3.5 s b) of corresponding initial (I), pretreated by NaOH solution (II), sonocatalytic (III), photocatalytic (IV), and photo/sono synergistically catalytic degradation of PET films (V), respectively. Weight loss c), FTIR‐ATR spectrum characterization d), and DSC analysis e) of PET films by the above different treated approaches. f) LC‐MS of the chemicals solution after the PET hydrolysis by NaOH solution. g–i) Time‐dependent H_2_, CO, and CH_4_ catalytic yields of MTSJA‐supported Z‐scheme heterojunction catalysts by photocatalysis, sonocatalysis, and photo/sono catalysis. j) H_2_, CO, and CH_4_ production rates of bare catalysts and MTSJA‐supported catalysts under photo/sono co‐catalysis approach.

PET, as a prevalently employed polymer, discharged into the sea, engenders substantial pressure to acquire drinking water. The reactive oxygen species technologies (e.g., enzyme catalytic, photocatalysis) are the most favored alternative to attack plastic, causing the fragmentation of macromolecules. Synergistically incorporating sound field with photocatalysis technology not only facilitates decomposing water molecules into the generation of hydroxyl radicals but also takes unparalleled advantage of the powerful penetrability of ultrasound waves. A thin aerogel is selected as an ideal platform to host the catalysts of SNCN/GQD@CdS for plastic degradation, and the optimal parameters of catalysts are explored by the degradation of methylene blue (Figure S12, Supporting Information). As the SEM and elemental mapping shown in Figure S13 (Supporting Information), catalysts have been successfully fabricated on MTSJA aerogel. The typical experiment of PET was cut into smaller films from a commercial PET film, which was exposed to 12 h catalytic activity under continuous AM 1.5 irradiation, a synchronous ultrasonic field with intermittent half‐hour action. The surface morphology of the original commercial PET is highly smooth before catalytic treatment and then appears massive regular folds on the surface of PET after being pretreated with NaOH solution (**Figure** **5**a(I,II). As shown in Figure 5a(III–V) of SEM images, it occurs drastic evolution of the surface morphology with the existence of abundant eroded indentations, fractured uneven fragments, rough surface, and the etching become progressively more severe from sonocatalysis to photo/sono catalysis, and it even forms microcracks in the insert of the enlarged image in Figure 5a(V). The pretreatment of alkaline environments enables a large number of OH^−^ in solution, which is favorable to the production of the active species of  OH for PET hydrolysis to cause the cleavage of macromolecules. The surface concavity pits facilitate internal corrosion by active species as much as possible and form “hot spots” for stress concentration, extending beyond the surface destruction into internal macromolecules to generate fragments and microcracks. The contact angle of the wettability evolution of PET is slightly decreased to 83.2° after alkaline treatment with an initial 87.1°, and is observed significant distinction after undergoing different catalysis approaches, in which the photo/sono catalytic PET shows maximum wettability (Figure 5b). It indicates the changes in surface roughness and surface free energy of PET as free radicals attack, further provoking the internal corrosion efficiency of PET by active species. It is worth noting that the weight loss of the PET obviously increases to 29.3% of photo/sono catalytic activity, in contrast to that of 24.1% and 27.7% by sono‐ and photo‐ catalytic degradation, respectively, for which the degraded matrixes of PET are responsible (Figure 5c). It can be seen from FTIR‐ATR spectra that all of the peak intensity declines of PET after different degradation treatments, accompanied by some shifts of individual peaks, such as the C‐H stretching vibration from 2920 to 2966 cm^−1^ (Figure 5d). It is associated with the essential variation of the internal molecular structure after degradation. In addition, there are slight rises in crystallinity of the degraded PET films read thermal information from the DSC characterization (Figure 5e), which can be calculated according to the Equation 4 expressed:^[^ ^21^ ^]^

(4) $$X_{c}=\frac{\Delta H}{\Delta H_{0}}\;=\frac{\Delta H_{m}-\Delta H_{c}}{\Delta H_{0}}\;\;$$

where Δ*H*_m_ means fusion enthalpy, Δ*H*_c_ signifies the crystallization enthalpy, and Δ*H*_0_ denotes enthalpy of complete fusion of corresponding PET of 140 J g^−1^. The crystallinity of the photo‐sono co‐induced degraded PET shows a slightly higher value of 69.67% compared with the initial PET of 51.82%, ultrasonic degradation of 58.11%, and photocatalytic PET of 64.44%. The increased crystallinities of PET certify the priority attack to the amorphous states by active species, with weakening intermolecular forces. Applying the same force with a weight to different degraded plastic films, the deepest cracks appear in photo/sono catalytic PET film (Figure S14, Supporting Information). The X‐ray photoelectron spectroscopy enables revealing the oxidation degree of the PET surface, which proves the oxidation evolution from the C─C bond to oxygen‐containing bonds, and the thorough discussion can be found in Figure S15 (Supporting Information). As shown in liquid chromatography‐mass spectrometry (LC‐MS) in Figure 5f, the major hydrolyzed products identified of PET undergoing pretreatment are some monomers, such as terephthalate (*m*/*z* = 165), ethylene glycol (*m*/*z* = 85), and isophthalate (*m*/*z* = 121). The peaks of 133, 143, and 181 could stem from oligomer fragments after PET hydrolysis. The solution degraded is diluted 50 times. To clarify the oxidative products of PET plastics, the liquid products are studied through HPLC and LC‐MS characterization (Figure S16, Supporting Information), and the gas products are deduced by photocatalytic gas production experiments by controlling CO_2_ sources input (Figure S17e, Supporting Information). Thus, the products of PET oxidation include liquid acetate, formic acid, lactic acid, etc., and minor gaseous H_2_, CO, and CO_2_. The in‐depth analysis of the oxidation mechanism of the PET matrix is revealed in Figure S16 (Supporting Information). The investigation also demonstrates the ethylene glycol monomer serving as the only sacrificial reagent from the photocatalytic PET oxidation process for upcycling fuel gas conversion.

#### 2.3.2 Photo/Sono Catalytic H_2_O‐CO_2_ Reduction

H_2_O and CO_2_ co‐transformation into clean fuel energy is attempted by synergistic photo/sono catalytic technology, in the case of PET/alkaline solution as a sacrificial agent for removing holes. The dual catalytic measurement was performed using a simulated photocatalytic system set up by an external portable ultrasonic shaker to simulate the ultrasonic waves, to prove the enhancement of photocatalytic activity by the ultrasonic cavitation effect. The photo‐, sono‐, photo/sono‐ catalytic hydrogen evolution performances of the MTSJA‐supported catalysts are monitored with reaction time changes (Figure 5g). The H_2_ yields of all of the samples present linear growth with the irradiation time, in which the photo/sono catalytic H_2_ production is observed the maximum slope to attest optimal reactivities. Consequences confirm the superior performance of photo/sono catalysis with achieving a value of H_2_ generation of 97.9 µmol g^−1^, which is 24.7% and 4.38 times enhancement than that of pure photocatalytic and sonocatalytic H_2_ production, respectively. It can be attributed to the following aspects. First, the excellent wettability of nanofibers endows the contact between active sites with water molecules decomposed into free radicals by the photo and ultrasonic energy. Afterward, CNTs adhering to the nanofibers potentially accelerate electron transfer, and the Z‐type heterojunction facilitates suppressing the recombination of electron–hole pairs. Besides, the Z‐type catalysts constructed are sensitive to mechanical waves, which is conducive to boosting the utilization efficiency of sonoluminescence.

The behaviors of CO_2_ transformation are surveyed with the main products of CO and CH_4_ detected (Figure 5h,i). Both CO and CH_4_ productivity display significantly improved results by photo/sono catalysis, such as showing the highest CO production performance of 64.3 µmol^−1^ g^−1^, almost 1.47 and 5.9 times the production from photo‐ and sono‐ catalytic CO_2_ reduction, respectively. The yields of producing CH_4_ are 9.8, 28.6, and 36.2 µmol g^−1^ of photo‐, sono‐, and photo/sono‐ catalysis. The catalytic rates demonstrate the aerogels‐based Z‐scheme heterostructures with greater efficiencies than pure catalysts, especially for CO_2_ reduction (Figure 5j). The lower yield of CH_4_ compared with CO results from the reactions producing CH_4_ needing greater surface charge density related to 8 electrons, which is four times the required electrons than the CO generation. In addition to the above reasons, the porosity structure of aerogel is beneficial to adsorb CO_2_ on the surface and improve contact with the active sites for activating the C─O bond. The profound mechanism of CO_2_ reduction will be elaborated upon in the next content.

It illustrates the impact of CO_2_ input on hydrogen production, shows the necessity of PET substrate, alkaline environment, and catalysts in gas production, and also evaluates the different performances of the PET, terephthalate, and triethanolamine substrate as sacrificial agents under 3 h photocatalysis yield (Figure S17a,b,e, Supporting Information). Combining minimal monitoring of CO and CH_4_ generation in the absence of CO_2_ injected (Figure S17e, Supporting Information), the isotope detection of the ^13^CO_2_ source demonstrates CO and CH_4_ mostly from CO_2_ and slightly from PET source. It also displays the gain in gas yield with the improvement of photo and ultrasound intensity. The mentioned above about more detailed discussions are presented in Figure S17, (Supporting Information). The contrastive morphological observation before and after photocatalytic measurements shows severe photo spalling of CdS from SNCN/CdS, conversely, there is no significant corrosion in the SNCN@GQD/CdS (Figures S18 and S19, Supporting Information). Accordingly, there are no signs of corrosion appearance on nanofibers of MTSJA, showing smooth form by the contrastive SEM images of before/after catalysis activity (Figure S20, Supporting Information). The metal Cd^2+^ concentration measurement in the aqueous solution of different aerogels‐supported catalysts after photo/sono catalysis is shown in Figure S21 (Supporting Information), which signifies the advantage of MTSJA to keep the catalytic activity and stability.

### 2.4 Photoelectrochemical Measurements and Proposed Mechanism

#### 2.4.1 Photoelectrochemical Performance

**Figure 6:**
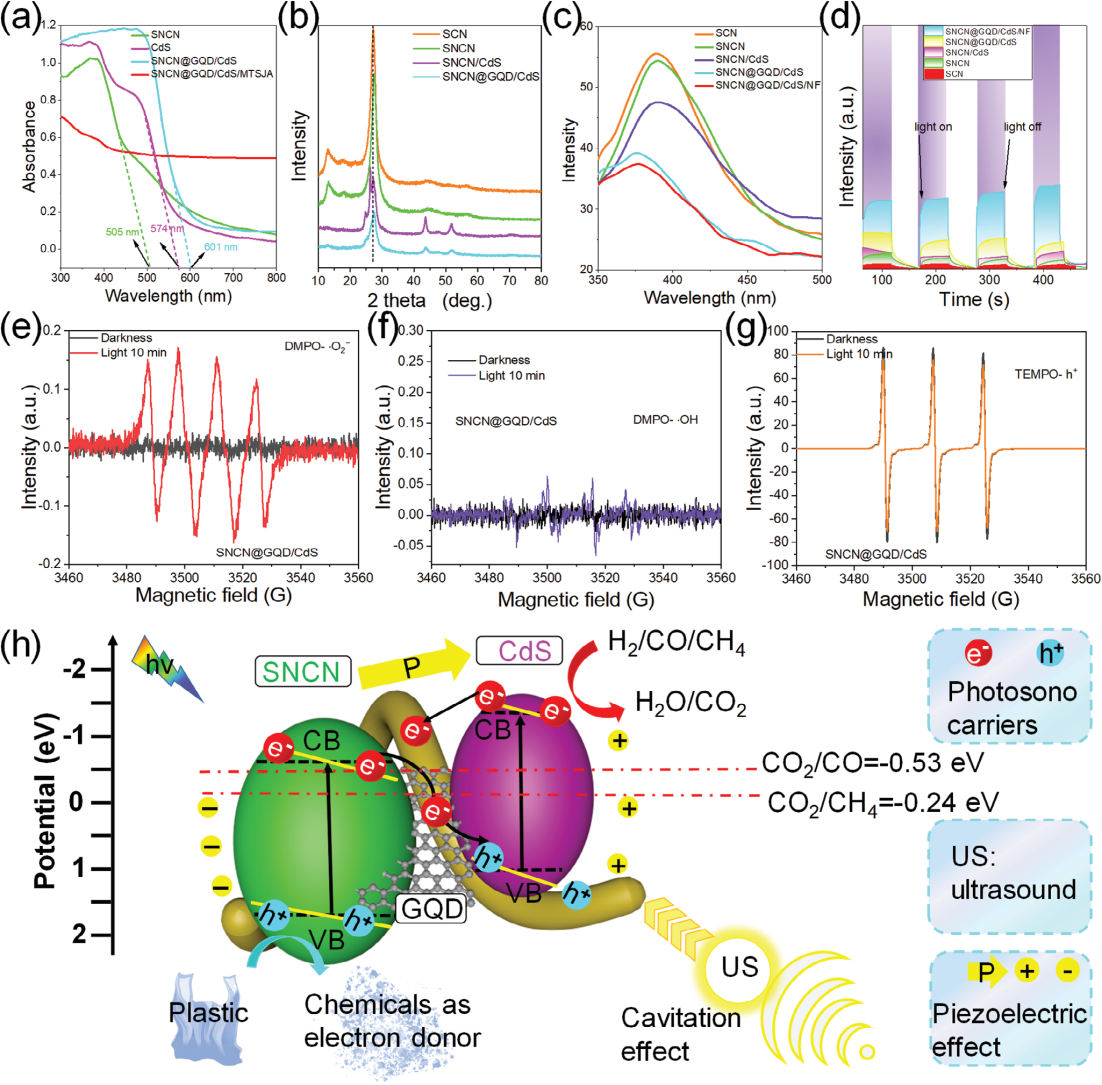
Analysis of photoelectric characteristics of SNCN, SNCN/CdS, SNCN@GQD/CdS, SNCN@GQD/CdS/MTSJA. a) UV–vis spectra. b) XRD patterns. c) Steady‐state photoluminescence spectrum with excitation wavelength at 320 nm. d) Time‐related photocurrent response of sample electrodes recorded in an electrolyte by light on/off cycles. ESR measurement of e) DMPO‐ O_2_^−^, f) DMPO‐ OH, and g) TEMPO‐ h^+^ before and after light irradiation. h) Schematically showing the possible synergistic photo/sono catalytic mechanism over MTSJA‐supported Z‐scheme heterojunction, for PET coupled with H_2_O‐CO_2_ high value‐added conversion.

UV–vis diffuse reflectance spectroscopy (UV–vis DRS) is depicted to analyze the light absorption capability (**Figure** **6**a). The absorption edge of SNCN is found at 505 nm, CdS displays a strong light capture within the range of 574 nm, and the nanocomposites of SNCN@GQD/CdS show a high light utilization range at 601 nm. The expansion of light absorption evokes photoproduced electrons. The SNCN@GQD/CdS loading on the MTSJA shows wider spectral absorption, owing to the catalysts and CNTs widening the spectral response (Figure S22a, Supporting Information). The XRD characterization of the phase composition and doping effects on crystal structure is analyzed of the diffraction peaks corresponding to these samples (Figure 6b). The results of sulfur‐doped g‐C_3_N_4_ (SCN) and SNCN present graphitic‐like diffraction peaks at ≈12.9˚ and ≈27˚ relevant (100) and (002) planes, denoting the in‐plane packing from tris‐s‐triazine motif and interplanar graphitic stacking reflection of π–conjugated aromatic systems, respectively.^[^ ^22^ ^]^ The (002) peak of SNCN shifts a higher diffraction angle accompanied by a relative intensity decrease than SCN, which demonstrates the decrease of the interplanar spacing in the c‐axis direction and the length of periodic stacking with particle sizes, respectively (Figure S22b, Supporting Information).^[^ ^23^ ^]^ It indicates a strong interaction of π–π stacked large aromatic supramolecular layer in the N‐doped construction process.^[^ ^24^ ^]^ Some strong diffraction peaks appear at 24.7˚, 26.67˚, 27.3˚, 43.59˚, 51.67˚ and weak peak of 47.8˚ (the purple curve), matching CdS crystal planes (PDF # 41–1049).^[^ ^25^ ^]^ The weakened diffraction peaks intensity of SNCN/CdS and SNCN@GQD/CdS crystal planes with significant changes, suggesting the CdS and GQD successful composite onto the surface of SNCN, especially for GQD with the π–π stacking interactions (Figure 6b). It is noted that the decrease in lattice distance due to a strong interaction between GQD and CdS provokes diffraction peak shifts from 27.3 to 27.44 (Figure S22c, Supporting Information). The low amount and low crystallinity of GQD are responsible for peaks with inconspicuous characteristics. To explore photoproduced carrier transfer in the NFs‐based catalysts system, NFs suspension containing CNTs mixed with catalysts was prepared, compared with other components of catalysts, for photoelectronic measurements. The photoluminescence (PL) spectrum is a common method for monitoring the activity of carriers, carried out under the excitation wavelength at 320 nm (Figure 6c). Generally, a lower PL signal denotes a more efficient carrier separation rate. It manifests SCN and SNCN with intense emission signals, suggesting rapid recombination rates of carriers. Compared with SNCN@GQD/CdS, NFs containing CNTs facilitate the enhancement of charge carriers transfer of SNCN@GQD/CdS. It is mainly associated with ladder‐functionalized electronic mediators of the GQD for forming a higher reduction potential of electrons, to greatly hinder charge recombination and accelerate the motion of charge carriers. Simultaneously, the recombination behavior of charge carriers is analyzed using the photocurrent test, showing a significantly increased photocurrent density of SNCN/GQD/CdS/NFs compared to other samples (Figure 6d).

The XPS spectra indicate the survey spectrum with C and N elements of CN, SCN, and SNCN, and S is not observed owing to the lower electron spectrum intensity (Figure S23, Supporting Information). The peaks at ≈287–288 eV are assigned to the sp^2^ s‐triazine ring (C─N).^[^ ^26^ ^]^ The binding energies of C 1s and N 1s shift, signifying the introduction of S and N altering the chemical environment in the SNCN. The binding energies of Cd 3d_5/2_ and S 2p in SNCN@GQD/CdS decrease to lower values compared to whether single‐component or two‐component, implying the electronic interactions of components and electron transfer from SNCN to CdS (Figure S24, Supporting Information). The FT‐IR analyzes surface functional group information of each component of catalysts (Figure S25a, Supporting Information). The EIS spectra show that the resistance radius of the SNCN/GQD/CdS/NFs is smaller compared to those other catalysts, testifying the optimal efficient separation and migration of photo‐sono co‐generated carriers in the Z‐scheme composite (Figure S25b, Supporting Information). Some free radicals of superoxide radical ( O_2_^−^), hole free radical (h^+^), and hydroxyl radical ( OH) are examined by adopting the ESR technology with the utilization of 5, 5‐dimethyl‐1‐pyrroline‐N‐oxide (DMPO) and 2,2,6,6‐tetramethylpiperidinyl‐1‐oxide (TEMPO) (Figure 6e–g). It is known that active species can also be confirmed through free radical capture experiments. Three scavengers of sodium oxalate (Na_2_C_2_O_4_), L‐ascorbic acid (LAA), and isopropanol (IPA) are added in the photocatalytic methylene blue (MB) degradation remove reaction to monitor h^+^,  O_2_^−^ and  OH, respectively (Figure S26, Supporting Information).^[^ ^27^ ^]^

#### 2.4.2 Possible Sono‐Photocatalytic Mechanism

Based on the aforementioned measurement and analysis results, it is necessary to attain insight into the charge transfer dynamic mechanism of the SNCN@GQD/CdS heterojunctions loading on the MTSJA system by ultrasound‐assisted photocatalysis (Figure 6h). The ultrasound‐induced piezoelectric performance is confirmed with insight discussion and the principles of ultrasound catalysis encompass as follows (Figure S27, Supporting Information). First, upon ultrasonic mechanical stress, the piezoelectric catalysts trigger the polarization effect to induce a relative displacement of charges, which results in an intrinsic piezoelectric field with the internal band tilting. Subsequently, the internal band deformation makes the flow of electrons and holes of CB and VB moving in opposite directions on the surface. Simultaneously, the collapse of ultrasonic‐driven cavitation bubbles evokes high pressures as high as 10^8^ Pa and a local “hot spot” upon 5000 °C in the interface of H_2_O and catalysts, to form heat‐excited water splitting to be converted into reactive species. Moreover, the difference in Fermi levels of SNCN/GQD@CdS and non‐centrosymmetric structures of SNCN and CdS are advantageous piezoelectric materials. In addition, the ultrasonic waves provoke a periodic mechanical driving force of catalysts to ensure reasonable piezometric potentials. The advantage of ultrasonic catalysis, combining lattice defects of oxygen and sulfur (Figures S22d and S24d, Supporting Information), offers a driving force for the generation of impetus to accelerate the charge separation. Finally, the synergistic existence of ultrasonic and photo fields is beneficial for the mass transfer of reactive radical species, particularly in targeting plastic wastewater which can be attacked by the powerful penetrability of ultrasound as a complementary role for photocatalysis.

Furthermore, the heterojunction system with a matching band structure plays a key role in catalysis, which can be inferred as conforming to the Z‐scheme mechanism. The VB potentials of SNCN and CdS are 1.91 and 1.03 eV, based on the bandgap values of 2.73 and 2.25 eV (Figures S28 and S29, Supporting Information), the CB potentials of SNCN and CdS are inferred with −0.82 and −1.22 eV versus NHE by the equation:^[^ ^28^ ^]^

(5) $$E_{\mathrm{VB}}=E_{\mathrm{CB}}\;+E_{g}$$

Consequently, under photo or ultrasonic radiation, electrons (*e* ^−^) are excited to transition on CB from VB of CdS and SNCN. The GQDs play a pivotal role in electronic media, which ensures the combination of *e* ^−^ on CB of SNCN with h^+^ VB of CdS availing on GQDs as conductive ladders, implying stronger oxidation potential (1.91 eV) and reduction potential (−1.22 eV). In addition, some electrons are possibly transferred onto the surface of CNTs with the probable attachment of catalysts and NFs.

The electrons accumulated on the CB of CdS develop H_2_O and CO_2_ reduction. The hydrophilic nanofibers are favorable for sufficient contact of H_2_O and Z‐scheme heterojunction. Adsorption and activation of CO_2_ serve as a prerequisite for CO_2_ reduction. Owing to the Lewis acidity of CO_2_, high porosity and large specific surface area of the nanofibers facilitate the large contact surface and capacity of CO_2_. Additionally, the polar functional groups of hydroxyl, carboxyl, and aldehyde in PVA‐*co*‐PE exhibit CO_2_ affinity, improving the mass transfer of CO_2_ and CO_2_ activity. CO_2_ molecules are induced the evolution from linear to curved structures by catalysis, bringing about reduced energy of LUMO of CO_2_ and reducing the activation energy barrier of CO_2_ with the production of ·CO_2_^–^. The enrichment of holes and high oxidation potential on the VB of SNCN are what propel the oxidation reaction of the plastic transformation. Benefiting from the degradation activity of PET to form the intermediate products, such as ethylene glycol and lactic acid as sacrificial agents, it achieves the generation of value‐added fuel of H_2_, CO, and CH_4_ from H_2_O and CO_2_.

(6) $$\mathrm{Catalyst}+\;h\nu +\mathrm{ultrasound}\to {e^{-}}_{\left(\mathrm{CB}\right)}\;+\;{h^{+}}_{\left(\mathrm{VB}\right)}$$

(7) $$h^{+}+\mathrm{PET}\to \mathrm{oxidation}\;\mathrm{products}\to \;CO_{2}+H_{2}O$$

(8) $$e^{-}\left(\mathrm{CB}\right)\;+H_{2}O\to H_{2}$$

(9) $$e^{-}\left(\mathrm{CB}\right)\;\;+\;\mathrm{oxidation}\;\mathrm{products}\;+\;CO_{2}\to \mathrm{CO}+H_{2}O$$

(10) $$e^{-}\left(\mathrm{CB}\right)\;+\;\;\mathrm{oxidation}\;\mathrm{products}+CO_{2}\to CH_{4}+H_{2}O$$

## 3 Conclusion

A complete floating aerogel‐supported Z‐scheme catalysts system is fabricated. The platform, with synergistic photo/sono technology, achieves high‐efficiency evaporation desalination and catalytic intermediate products from PET degradation promoting H_2_O‐CO_2_ conversion. The aerogel demonstrates superior desalination by the functions of excellent air–water interface photothermal, thermal resistance barrier with water bodies, water supply, and resonance effects between ultrasound waves and salt crystals, with the consequences of the evaporation rate of 3.1 kg m^−2^ h^−1^ of 82.3% efficiency under 21 wt.% NaCl solution at one sun irradiation. Meanwhile, the compelling catalytic performance of PET, H_2_O, and CO_2_ coupled value‐added transformation depends on the amalgamation: ultrasonic cavitation effect, mechanical wave sensitivity, and electron mediator of GQDs of the Z‐type SNCN@GQDs/CdS catalysts, which boosts the synergistic effects of photo/sono. Thus, it realizes the photo/ultrasound catalytic fuel transformation from H_2_O and CO_2_ into H_2_, CO, and CH_4_ yields with 16.1, 9.5, and 3 µmol h^−1^ g^−1^, utilizing plastic as the reaction substrate into sacrificial agents to facilitate photo/sono catalytic half‐reaction. This work may inspire the thought of 2D interfacial evaporation and energy harvesting and transformation utilization of solar and marine sonar. It also opens perspectives to develop waste (e.g., plastics and textiles) upcycling into innovations for clean fuel acquisition.

## 4 Experimental Section

### Chemicals and Materials

Copolymerized polyvinyl alcohol and polyethylene (PVA‐*co*‐PE) masterbatches contain 44 wt.% PE, showing the molecular weight of the copolymer is ≈70 000 to apply (F101B, Kuraray Co., Ltd., Japan), cellulose acetate butyrate (CAB, 381–0.5, Eastman Shanghai chemical Commercial Co., Ltd). Carboxyl multi‐walled carbon nanotubes were prepared (CMCNTs, 0.5–2 µm, hydroxylation content 5.58%, J&K Scientific Co., Ltd., China). Methyl trichlorosilane (AR), hydrochloric acid (HCl), glutaraldehyde, acetone, and isopropanol were obtained from Sinopharm Chemical Reagent Co., Ltd., China. Graphene quantum dots (GQD, 100 mL, 1 mg mL^−1^) were purchased from Nanjing XFNANO Materials Tech. Co., Ltd., China. In addition, most of the chemical reagents were supplied by Shanghai Macklin Biochemical Technology Co., Ltd., China, including thiourea, cadmium nitrate tetrahydrate (Cd(NO_3_)_2_ 4H_2_O), thioacetamide (TAA), sodium dodecyl sulfate (SDS), caustic soda (NaOH), methylene blue (MB).

### Fabrication Procedure

*Preparation of BBLA*: For the preparation of BBLA, it was necessary to preferentially fabricate the PVA‐*co*‐PE nanofiber suspension that was derived from the traditional craftsmanship of the group.^[^ ^29^ ^]^ First, PVA‐co‐PE/CAB hybrid fibers were obtained by the molten extrusion technology, and the CAB was removed with acetone through the phase separation principle. The PVA‐*co*‐PE nanofiber suspension dispersing in water and isopropanol (mass ratio of 1:1) was fabricated by a high‐speed shearing approach. Second, the nanofibers suspension was cross‐linked for 1 h with glutaraldehyde as a cross‐linking agent and HCl as a catalyst and poured into a 3D‐printed mold for freeze drying. A “sunken tenon” structural aerogel with hydrophily was acquired.

Afterward, the hydrophobic “sunken tenon” structural aerogel was achieved by a facile chemical vapor deposition according to the previous report.^[^ ^30^ ^]^ In detail, methyl trichlorosilane ≈5 mL was used as a hydrophobic modifier, and the as‐prepared hydrophilic aerogel was placed at a certain suspended height in a sealed reactor. The modification reaction was provided with a condition at 60 °C for 12 h. To clean up methyl trichlorosilane for subsequent use, the as‐prepared hydrophobic aerogel was vacuum‐dried.

*Preparation of ULLA*: Additionally, carboxyl‐modified CNTs were subjected to cell disruption for 2 h. The PVA‐*co*‐PE nanofiber suspension and CNTs solution (0.1 wt.%) were mixed in a ratio of 10:1. The mixture was cross‐linked for up to 2 h using a cross‐linking agent: glutaraldehyde: hydrochloric acid in a ratio of 10:1:0.1. To fabricate a “protruding mortise” structure firm joining “sunken tenon”, the tenon aerogel was soaked in 30 wt.% isopropanol for a period of time before pre‐freezing, resembling a honeycomb mold. The PVA‐*co*‐PE‐doped CNTs suspension was poured into the above pre‐freezing honeycomb mold for further drying to form a novel “mortise‐and‐tenon structure” Janus aerogel with the fully floating function.

*Preparation of Z‐Scheme SNCN@GQD/CdS Heterojunction Catalysts*: First, thiourea and citric acid powder (mass ratio of 1000:1) were placed in a porcelain boat under evenly grinding and annealed at 550 °C under air with a rate of 2.5 °C min^−1^ for 4 h, acquiring sulfur‐and‐nitrogen doped graphitic carbon nitride (SNCN). Typically, 0.4 g of SNCN power was added into 30 mL of deionized water under magnetic stirring and sonicated exfoliation for 30 min. 1 mL GQD dispersion liquid, 0.024 g of Cd(NO_3_)_2_ and 0.0078 g of TAA power were mixed with 10 mL pure water, which were sonicated for 30 min to achieve well mixed. Subsequently, the mixture solution underwent hydrothermal synthesis at 160 °C for 6 h after being transferred into a 60 mL autoclave.

*Preparation of Aerogel‐Supported Z‐Scheme Catalysts*: The catalysts were modified by a certain concentration of SDS at 100 °C for 12 h and poured after centrifugation at 4000 rpm for 5 min. Then, the catalysts collected were deposited on the aerogel under 60 °C for 2 h.

### Photo/Sono Evaporation Desalination and Catalysis Measurement

*Photo/Sono Evaporation Desalination Measurement*: The as‐prepared aerogel was placed on water surface in beaker based on its self‐floatable performance. The evaporation environmental temperature and humidity were controlled by the air conditioner and humidifier to keep at ≈25 °C and humidity of ≈45%. The evaporator was placed in the central area of a simulated solar system with a 300 Xenon lamp with the standard AM 1.5 spectrum with a 420 nm cut‐off filter under 1 solar (1 kW m^−2^) radiation for 30 min. A typical measurement was the infrared thermal imager and laboratory balance used to real‐time monitor the surface temperature evolution of aerogel and water mass loss at every minute. The salt‐depositing evaporation measurement was repeated the above test under an external and portable ultrasonic shaker irradiation with 70 W or photo‐*co*‐ultrasonic irradiation.

*Photo/Sono Catalytic MB Degradation for the Optimal Parameter*: First, the optimal parameters of aerogel‐supported catalysts can be confirmed by photocatalytic degradation of MB. The MB solution (100 mL, 10 mg L^−1^) combined with aerogel‐supported catalysts was ultrasonic pretreated in the darkroom for 30 min to reach adsorption equilibrium. There was a little alteration compared with the evaporator that thinner aerogel was suitable for catalysis. The suspension was extracted 2 mL once per 10 min and tested the absorption changes by UV–vis spectrophotometer. Then, the degradation process and degradation efficiency were calculated.

*Photo/Sono Catalytic Plastic PET Conversion*: To ensure the consistency of the PET‐based substrate, a film with a thickness of 200 µm was employed and cut into smaller fragments. PET (20 mg mL^−1^) fragments were added in 10  NaOH to maintain stirring pre‐treatment for 24 h. Then, the aerogel‐supported catalysts were placed on the above solution for the degradation test under a similar type of light source and ultrasonic field with the degradation of MB, while modifications of irradiation were up to 12 h. It was worth noting that the ultrasonic working mode was every 30 min on/off, signifying the actual ultrasonic working time with 6 h. After degradation, the solid residue including PET fragments mixtures was collected by Buchner funnel with 0.1 µm nanofiber membrane filter after washing, and the organic compounds degraded in the solution were further analyzed.

*Photo/Sono Catalytic H_2_O‐CO_2_ Conversion*: After pre‐treatment to plastic PET fragments by NaOH solution, the PET‐bottle‐based aqueous solution was collected as the catalytic reaction substrate. Then, the composite aerogel catalysts were put on the above solution in a quartz reactor. CO_2_ was insufflated into the reactor and kept adsorption equilibrium with the porous aerogel in darkness. An ultrasonic shaker was put in another external quartz, and the light source was adjusted at a certain angle for illumination. All gases were recorded by gas chromatography at 1 h intervals.

## Conflict of Interest

The authors declare no conflict of interest.

## Author Contributions

Y.L. and T.Y. contributed equally to this work. Y.L. supervised, conceived the idea, designed the entire project and wrote the manuscript. T.Y. and Y.W. performed the experiments and data analysis. J.C., H.Y., and J.L. assisted the data analysis. Y.X. and Z.X. took part in characterizing partial measurements. J.L., Y.Q. contributed the results analysis and mechanism discussion. W.W. and D.W. provided experimental resources and financial support.

## Supporting information

Supporting Information

## Data Availability

The data that support the findings of this study are available in the supplementary material of this article.
