# Supplementary material for: Fully Floatable Mortise‐and‐Tenon Architecture for Synergistically Photo/Sono‐Driven Evaporation Desalination and Plastic‐Enabled Value‐Added Co‐Conversion of H2O and CO2
Source: Adv Sci (Weinh). 2024 May 20;11(29):2404423. doi: 10.1002/advs.202404423 (PMC11304291; doi:10.1002/advs.202404423)
Supplement: Supplementary file 1 — Supporting Information [file ADVS-11-2404423-s001.docx]

# Supporting Information

# Fully Floatable Mortise-and-Tenon Architecture for Synergistically Photo/Sono-Driven Evaporation Desalination and Plastic-Enabled Value-Added Co-Conversion of H_2_O and CO_2_

*Yingying Li*, Tongrong Yao, Yanqiu Wang, Jiahui Chen, Haining You, Jing Lu, Yi Xiong, Zhongduo Xiong, Jia Liu, Yajuan Qi*, Wenwen Wang, and Dong Wang**

Dr. Y. Li, T. Yao, Y. Wang, J. Chen, H. You, J. Lu, Y. Xiong, Z. Xiong, W. Wang, Prof. D. Wang

Key Laboratory of Textile Fiber and Products, Ministry of Education, Wuhan Textile University, Wuhan 430200, China

E-mail: yyli_opt@126.com; wangdon08@126.com

Dr. J. Liu

Multifunctional Electronic Ceramics Laboratory, College of Engineering, Xi’an International University, Xi’an, 710077, China

Dr. Y. Qi

College of Science, Wuhan University of Science and Technology, Wuhan 430081, China

E-mail: yajuanqi@wust.edu.cn


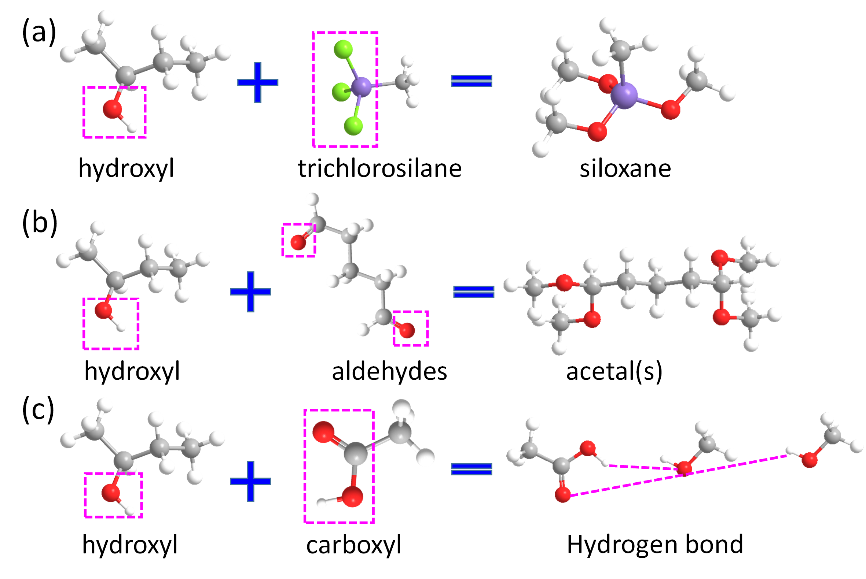


**Figure S1.** Schematic diagram of the chemical evolution of MTSJA preparation of BBLA (a) and ULLA (b-c).


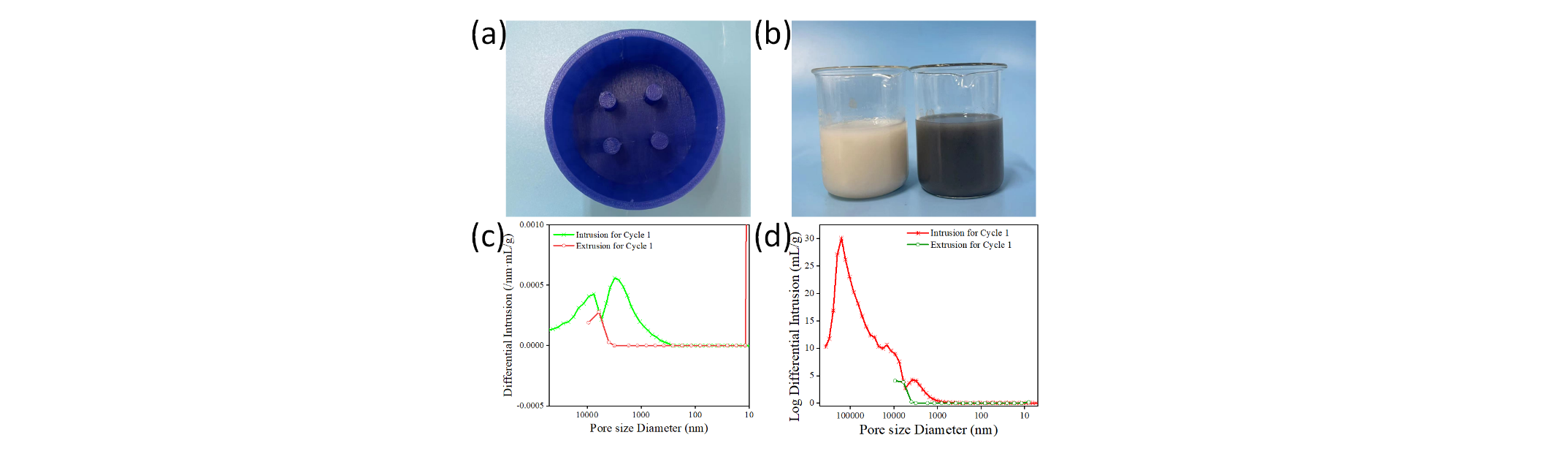


**Figure S2.** Digital photographs of 3D printing mold of preparing BBLA (a) and the initial PVA-*co*-PE commercial nanofibers suspension and commercial nanofibers doped CNTs suspension (b). The porosity measurement of the MTSJA aerogel by the mercury intrusion porosimetry (c-d).

SDS enhancing the performance of the catalysts, such as preventing caking catalysts on the surface of aerogel, improving the stability of catalysts, and high affinity between catalysts and H_2_O molecules.


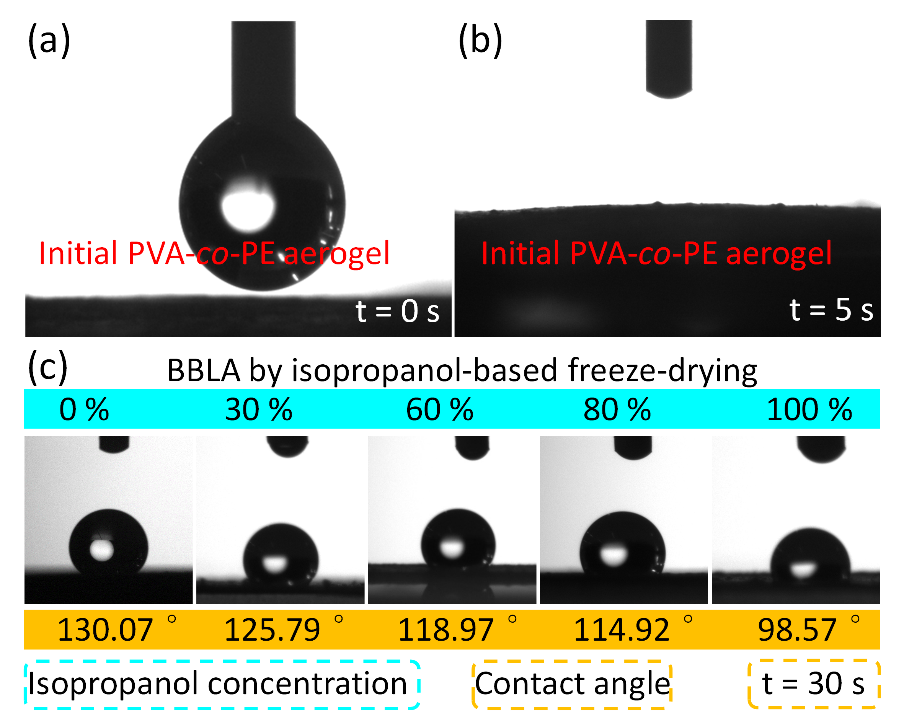


**Figure S3.** The photographs displaying surface wetting changes of samples. (a-b) The contact angles of initial PVA-*co*-PE aerogel. (c) The different contact angles of the BBLA samples undergoing different concentrations isopropanol immersing and freeze-drying treatment.

According to the experimental sequence, the initial PVA-*co*-PE aerogel is preferentially prepared, showing a small dynamic contact angle during a short period interval (Figures S3a-S3b, Supporting Information). Afterward, BBLAs are endowed with different concentrations of isopropanol freeze-drying treatment and exhibit gradient-reduced contact angles with increasing concentrations of isopropanol (Figure S3c, Supporting Information).


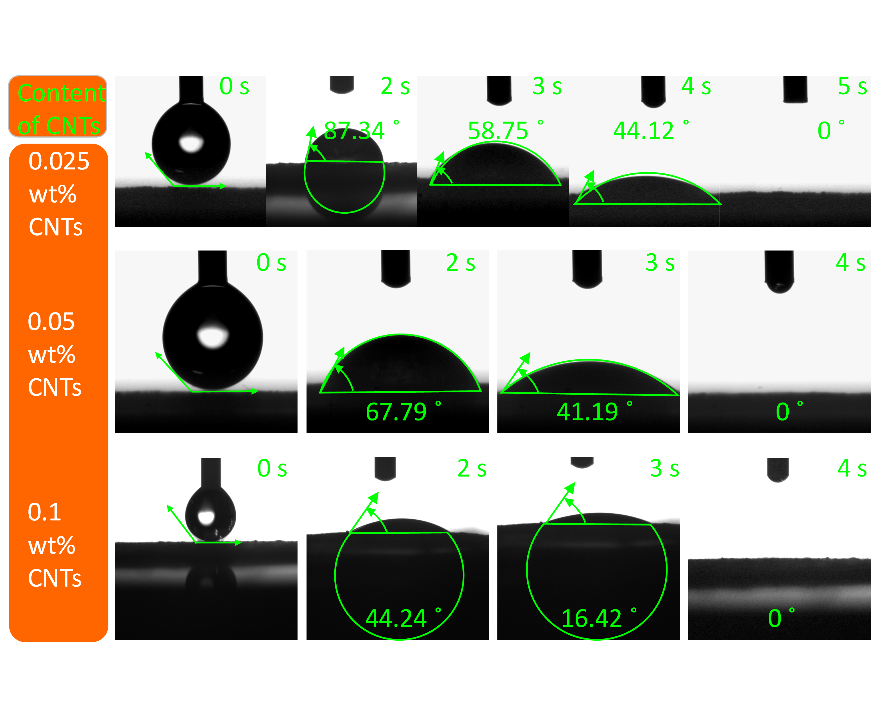


**Figure S4.** The contact angles characterization of ULLA. The photographs showing the surface wettability increase of ULLA as the doped content increase of CNTs of 0.025 wt% (a), 0.05 wt% (b), and 0.1 wt% (c).

It is deduced BBLA with a relatively great wettability to isopropanol, and the polar property of isopropanol enhances the interaction with silane bonds and surface energy of aerogel for subsequent construction of ULLA. For ULLA, it can be noticed a significant increase in wettability with the addition of CNTs (Figure S4, Supporting Information).


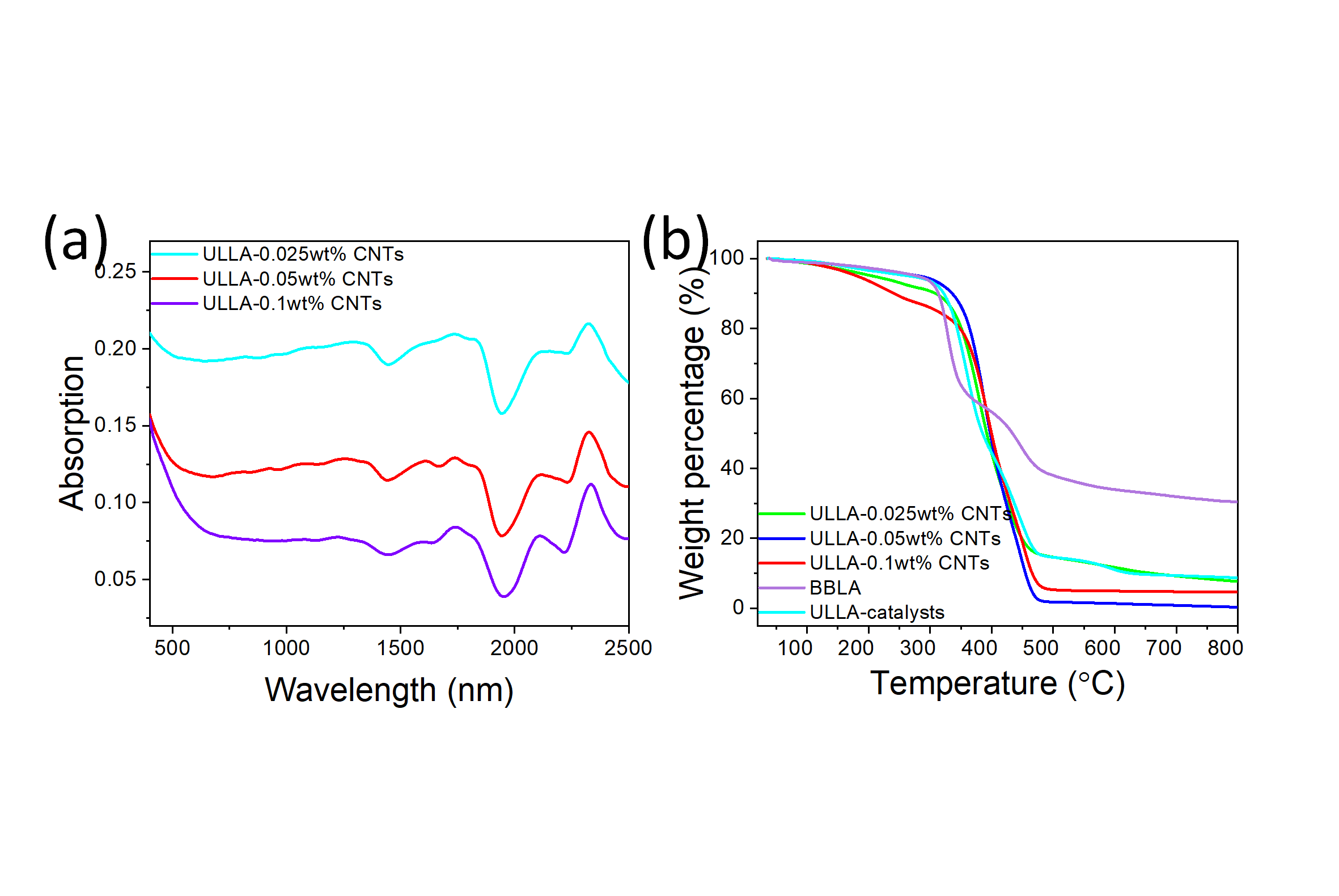


**Figure S5.** (a) UV-vis-IR spectrum characterization of ULLA with different amounts of CNTs. (b) TGA of ULLA, BBLA, and ULLA-supported catalysts.


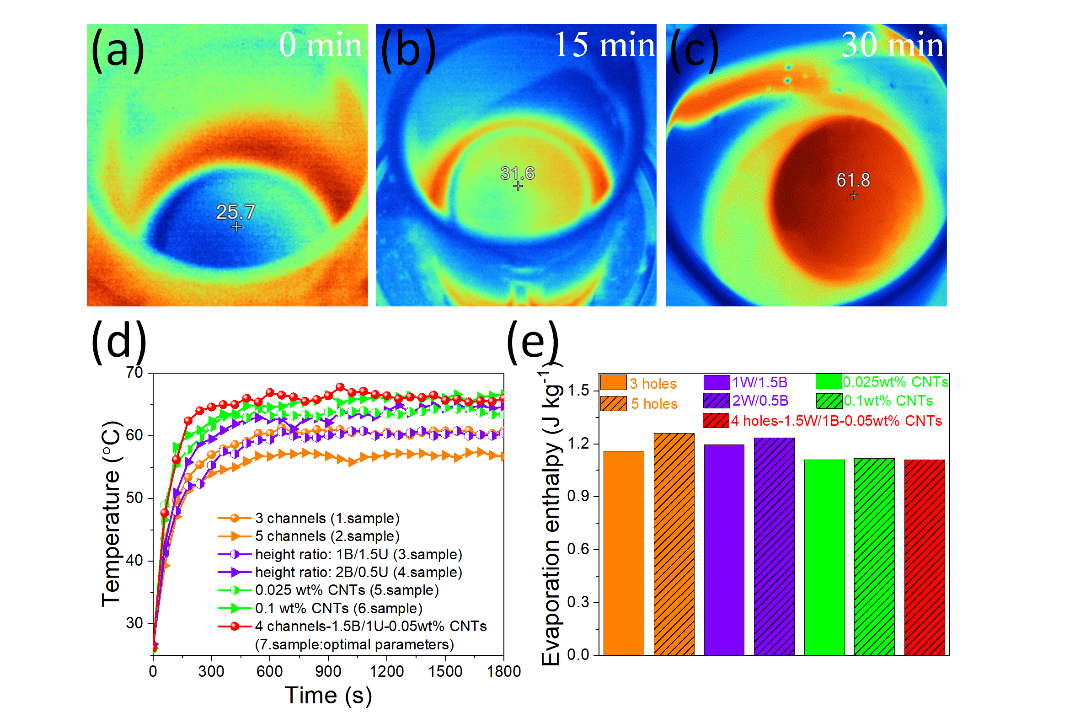


**Figure S6.** Solar-driven evaporation of MTSJA. (a-c) The IR images of surface temperature changes of MTSJA recorded by infrared photography under solar irradiation of 30 min. Surface temperature evolution as a function of time (d) and calculated dark equivalent enthalpy (e) of the MTSJA by regulating the numbers of hydrophilic channels, height ratio between BBLA and ULLA with a constant total height, and the contents of CNTs in the ULLA.

Infrared images reveal an intuitive photothermal conversion phenomenon of the optimal evaporator in the steam generation process (Figures S6a-S6c, Supporting Information). The surface temperature of MTSJA ascends and then descends as the increase of hydrophilic channels and the height ratio of BBLA/ULLA (Figure S6d, Supporting Information). The highest contents of CNTs show maximum temperature difference change. It can be seen that the optimal aerogel could significantly lower the evaporation enthalpy (Figure S6e, Supporting Information).


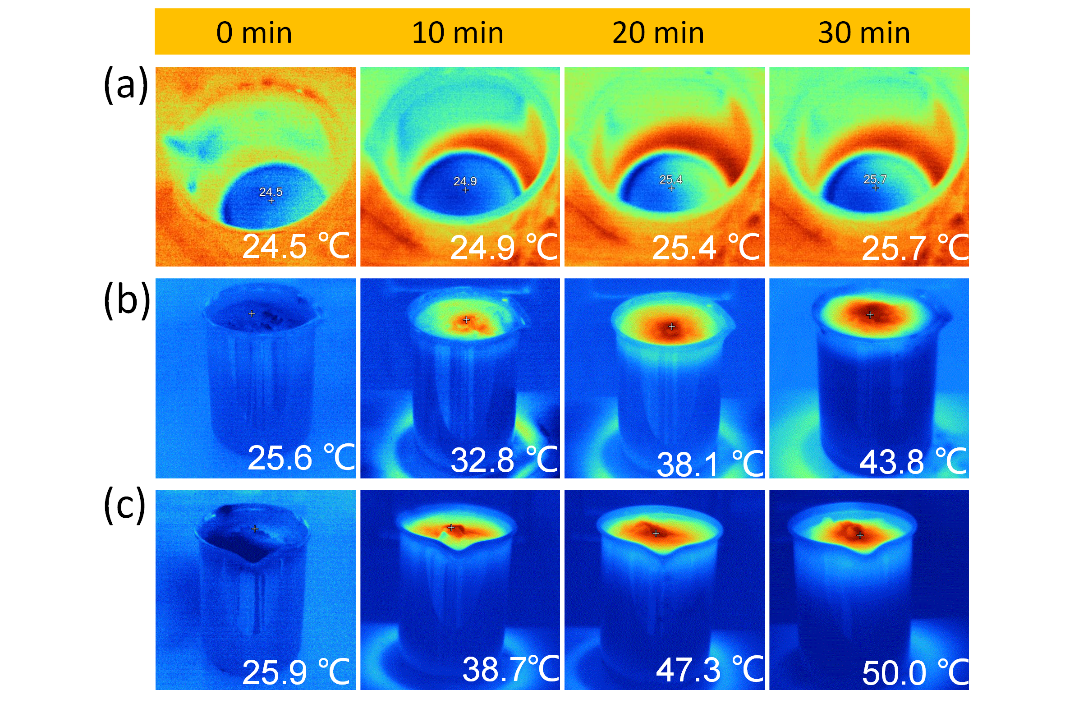


**Figure S7.** The infrared thermal imagery of surface temperature evolution of MTSJA evaporator, showing pure ultrasound (a), photo (b), and photo/sono (c) evaporation desalination under 30 min irradiation of 30 min in 21 wt% NaCl solution.

The thermal effect of ultrasonic waves can be negligible based on the low thermal conductivity of aerogel (Figures S7a-S7b, Supporting Information), and it displays the thermal localization surfaces of the MTSJA evaporator without thermal conduction into water bodies in Figure S7c, Supporting Information.


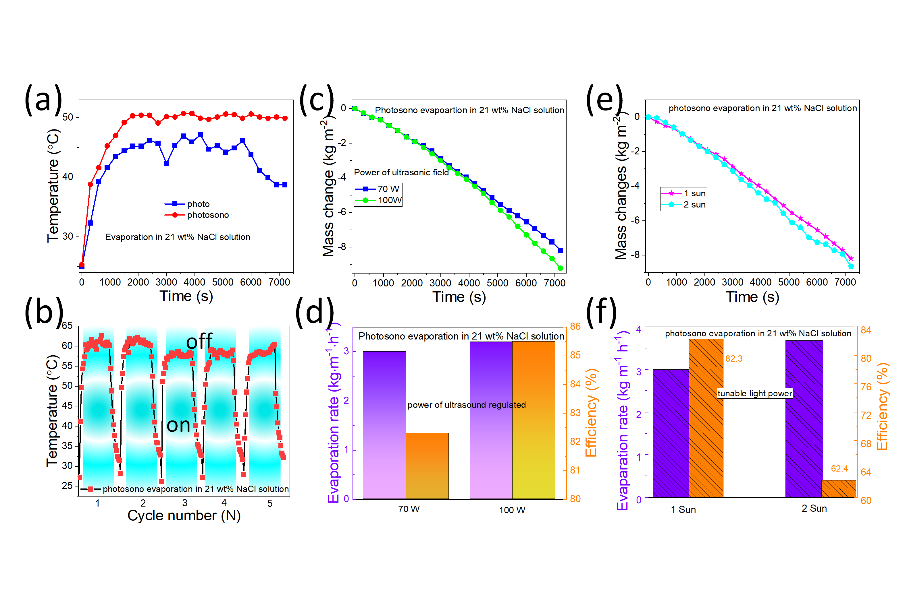


**Figure S8.** Solar-sono-enabled evaporation desalination of MTSJA. (a) Surface temperature evolution as a function of time of photo and photo/sono driven desalination in 21 wt% NaCl solution. (b) Heating-cooling behavior of the MTSJA under multiple cycles with light on and off in 21 wt% NaCl solution. The mass change, evaporation, and efficiency of evaporation desalination of MTSJA under regulating ultrasound power (c-d) and solar power (e-f).

It shows the enhanced photothermal conversion at the photo/sono field compared with pure photo irradiation in long-term evaporation because of few salt accumulations (Figures S8a-S8b, Supporting Information). Further photo/sono-driven evaporation desalination is tested by controlling solar or ultrasound powers (Figures S8c-S8f, Supporting Information). It indicates the higher evaporation rate and efficiency as the increase of ultrasound powers, but under two sun irradiation despite the evaporation rate rise, the efficiency still reduces. It demonstrates the hazard of salt accumulation.


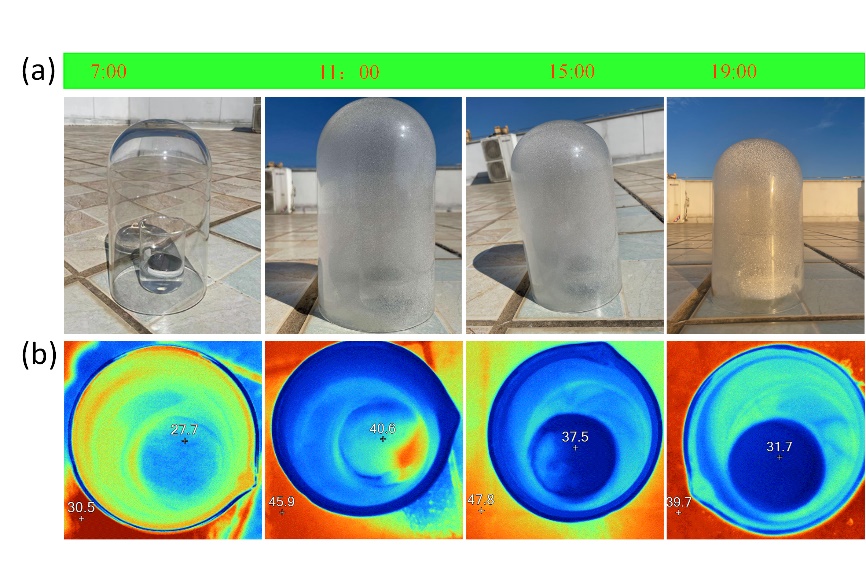


**Figure S9.** Outdoor evaporation of MTSJA. The photographs of outdoor evaporation within a 4-hour time interval (a), and the corresponding infrared thermal images (b).


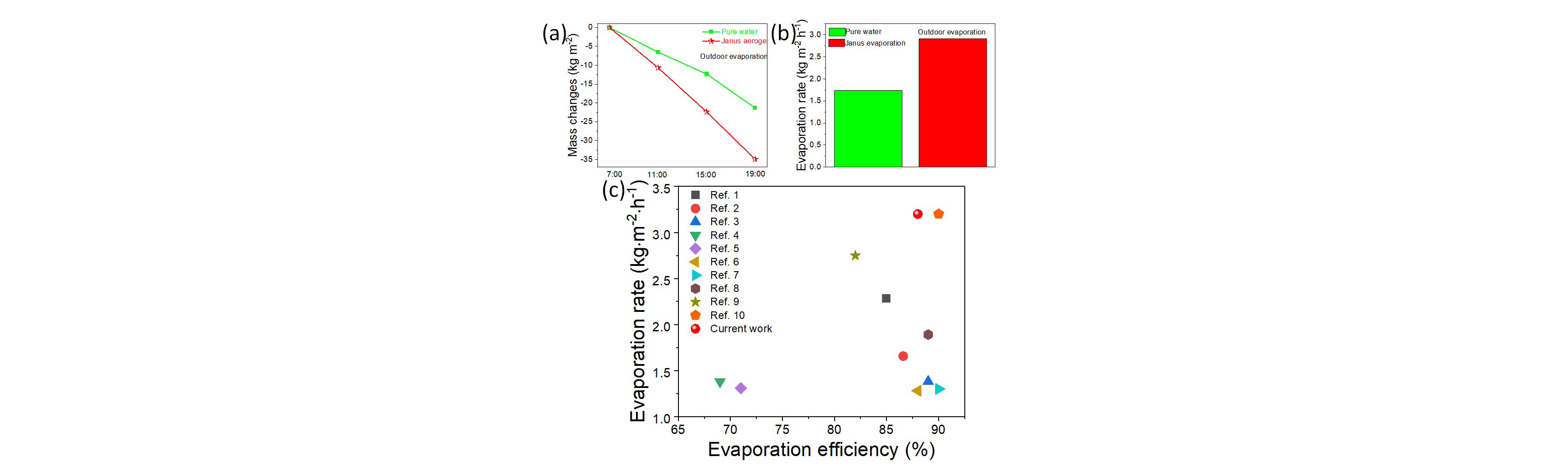


**Figure S10.** The mass changes (a) and evaporation rate (b) of the outdoor evaporation of MTSJA. (c) Comparison of the MTSJA evaporation rate and efficiency and previously reported performance under 1 sun.


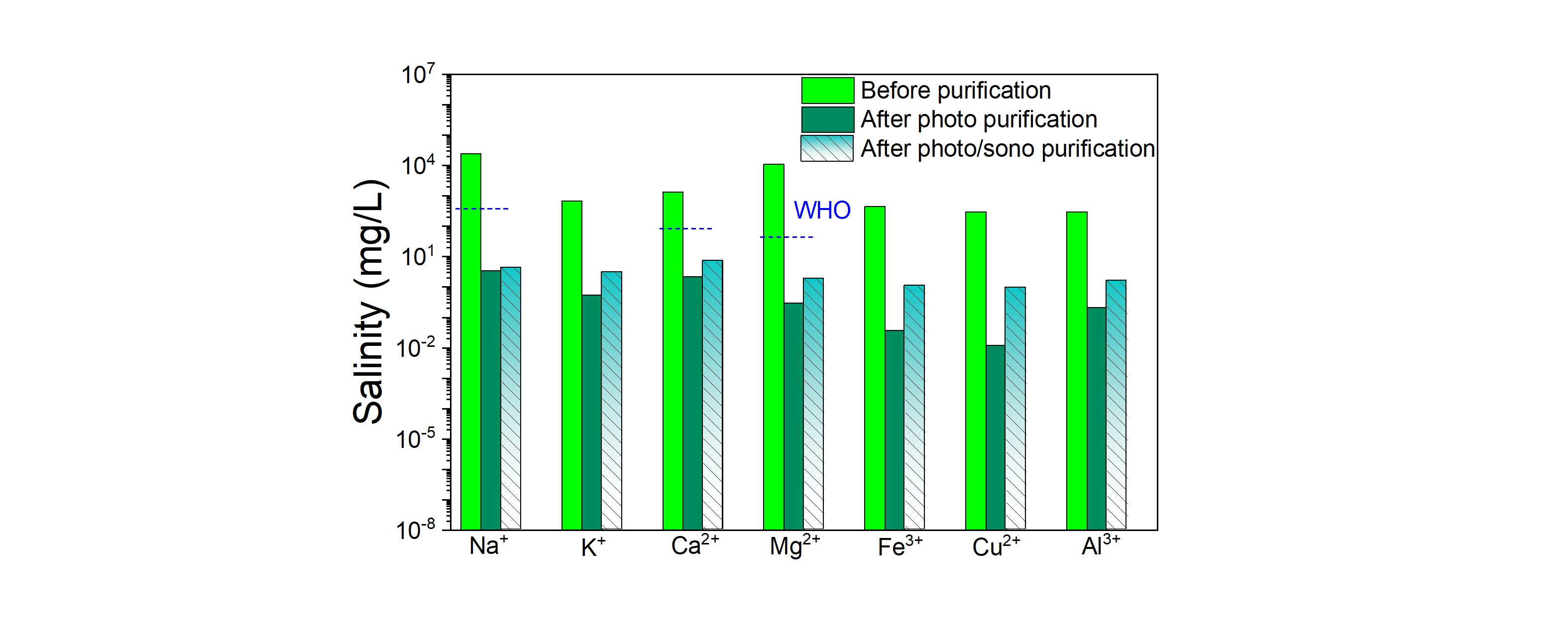


**Figure S11.** The comparison of the concentrations of multiple metallic ions in seawater before, photo, and photo/sono desalination.


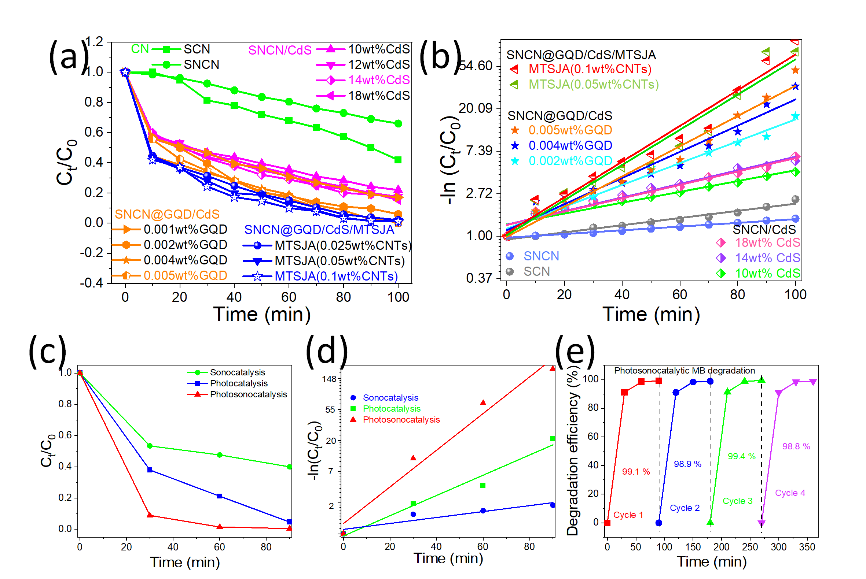


**Figure S12.** The photo/sono catalytic degradation of methylene blue. The photocatalytic degradation efficiency (a) and corresponding variations in –ln (C_t_/C_0_) (b) of methylene blue of SNCN@GQD/CdS/MTSJA by adjusting the different parameters of SNCN, GQD, CdS, and MTSJA. The degradation efficiency (c) and corresponding variations in –ln (C_t_/C_0_) (d) by photo, sono, and photo/sono catalytic technology. (e) The cyclic degradation of methylene blue by photo/sono catalysis.


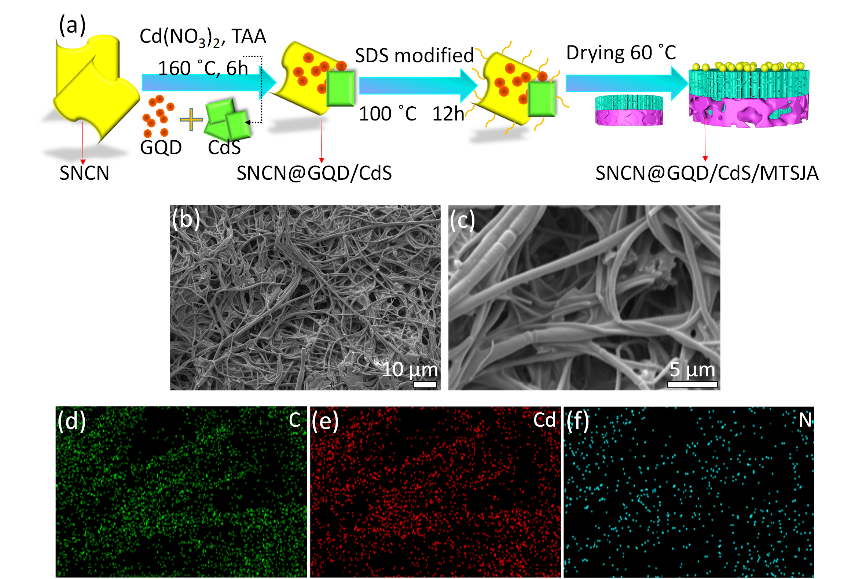


**Figure S13.** The structure characterization of SNCN@GQD/CdS/MTSJA. (a) The schematic of the SNCN@GQD/CdS/MTSJA nanocomposites. The SEM images of SNCN@GQD/CdS loading on MTSJA (b), the corresponding magnified images (c), and the EDX elemental mapping images of C, Cd, and N of the interface of MTSJA-supported SNCN@GQD/CdS (d-f).


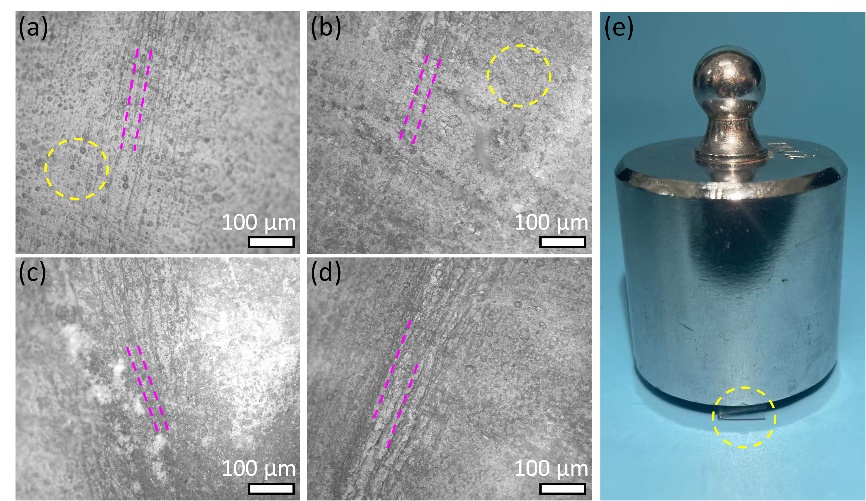


**Figure S14.** The optical microscope images of cracks induced by stress with the same weight on different PET films after undergoing initial (a), sono catalytic (b), photocatalytic (c), and photo/sono catalytic (d) treatment. (e) The digital photographs of 200 g weights applied to the different films.

The gas phase of PET film after catalytic treatment reduces compared with the initial PET plastic, being beneficial for the production of smaller fragments to facilitate the decomposition of PET.


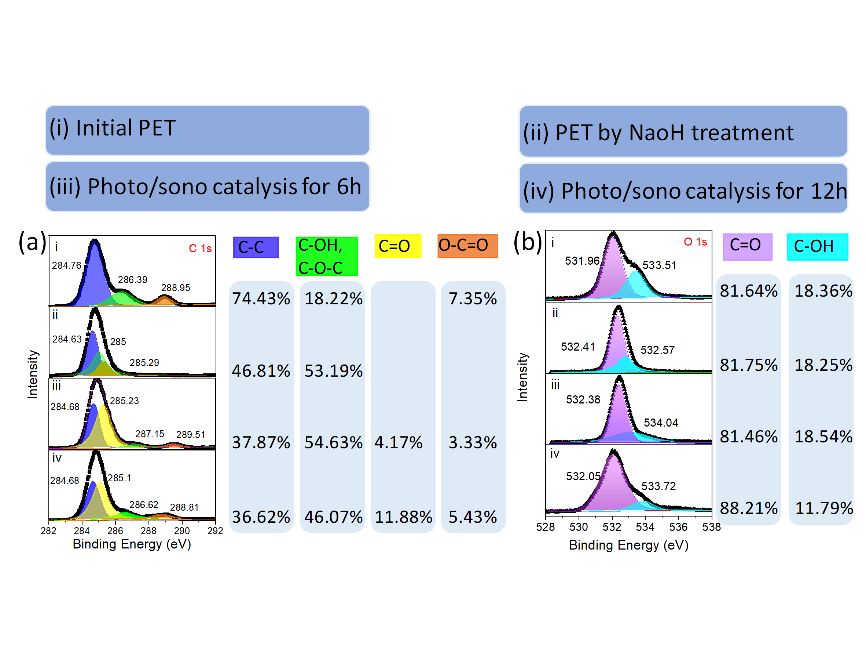


**Figure S15.** XPS survey of (a) C 1s and (b) O 1s peak deconvolution of (i) initial PET, (ii) pre-treated PET, (iii) photo/sono catalytic PET oxidation for 6 h and (iv) photo/sono catalytic PET oxidation for 12 h.

The X-ray photoelectron spectroscopy enables the revealing of the oxidation degree of the PET surface, especially the C/O content ratio as a weathervane of oxygen-containing bond evolution, such as C=O and C-OH on the PET surface. The XPS high resolution detected the decrease of carbon content along with the increase of oxygen. In the C1s spectrum (Figure S15a, Supporting Information), the peaks situated about 285 eV, 286 eV, and 288-289 eV are respectively classified as the methyl group, glycol C-O-C group, and ester or carboxyl groups of C=O/O-C=O. The O1s (Figure S15b, Supporting Information) at about 531 eV and 532 eV involve the C=O and C-OH groups. After NaOH and catalysis treatments, the C-C peak gradually transitions into C=O. The degradation mechanism possibly is preferential against surface erosion inducing the cleavage of chemical bonds. The chemical bonds of the PET are excited to the excited states (LUMO) by the photo/sono catalysis, which undergoes the redox reaction with free radicals of ∙O_2_^-^ and ∙OH from the catalysts. Ultimately, it brings about the creation of C=O and O-C=O groups. It C/O ratio changes implies that the enhanced affinity for water of the degraded PET film, supporting the contact angle analysis in Figure 5b. Due to the insensitivity of the benzene ring to catalysis, ethylene glycol denoting another PET hydrolyzate at the terminal end of the carbon chain with the C-C bond, is susceptible to oxidation into ester or carboxyl groups (C=O). Thus, combining the FTIR, the PET oxidation process may undergo the transformation of methylene and begin the C-C bond scission to form the C-O bond, and oxidation production of O-C=O, C=O.


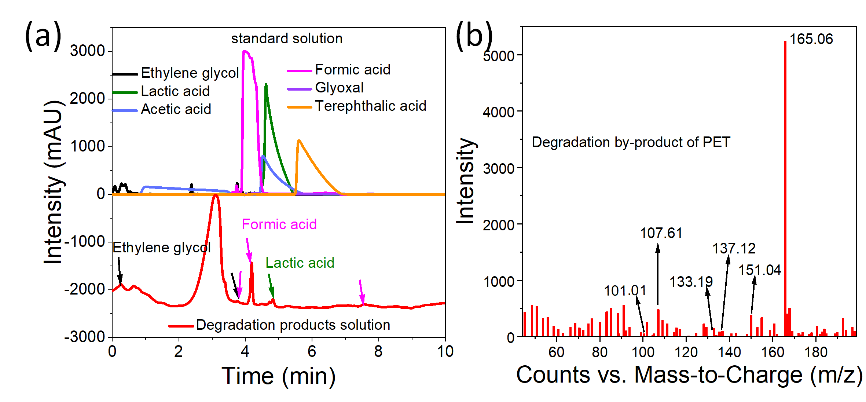


**Figure S16.** (a) High-performance Liquid Chromatograph (HPLC) and (b) Liquid chromatography-mass spectroscopy (LC-MS) of the chemicals from the PET degraded solution by photo/sono catalysis.

*The* *Mechanism of PET Substances Oxidation Products.* It involves adsorption-activation and oxidation in the photo/sono reforming EG (ethylene glycol). The EG molecules are initially adsorbed onto the catalysts and nanofibers surface and activated. Then, there are two possible forms of oxidation, typically signifying direct oxidation of EG monomer by holes of VB or indirect oxidation ways by ∙OH free radical. As well known, the TPA (terephthalate) monomer can act as a scavenger of ∙OH. Thus, the oxidation of EG substrates primarily occurs through hole excitation.

Besides, the product pathways may involve the following mechanism, including liquids and small amounts of gases, according to the control experiments of gas products and XPS, HPLC, LC-MS, and isotope characterization (Figures S15-S17, Supporting Information). (i) The active sites of the catalyst facilitate the activation and dehydrogenation of hydroxyl groups in EG, to form acetaldehyde, which is further oxidated into acetic acid, formic acid et al liquid products. (ii) Gases products: ∙CH_3_ free radicals from acetic acid undergoing decarboxylation behavior can combine with H^+^ to form CH_4_ gas; Moreover, EG gradually occurs oxidation formation glycolaldehyde and glycollic acid, as well as decarbonylation into methyl alcohol which sequentially generates CO. (iii) In the steps, the dehydrogenation reactions of EG and methyl alcohol, and the decarboxylation reactions of acetic acid result in the loss of protons in PET and hydrogen production from EG substrate. Therefore, the source of protons of H_2_ partially originates from the precursor EG with small parts, while the majority result from water.


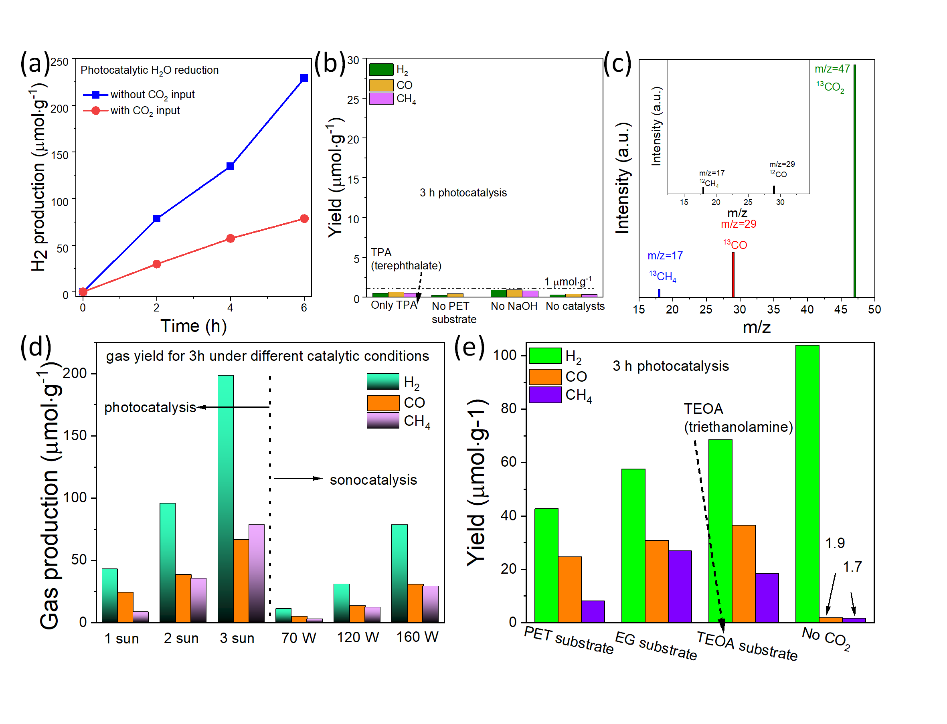


**Figure S17.** Photo and sono catalytic capabilities under different influence factors. (a) Photocatalytic H_2_ evolution by contrast of CO_2_ input and CO_2_-free input. (b) Comparison of photocatalytic gas production of H_2_, CO, and CH_4_ (for 3h) with different conditions of only TPA, no PET, no NaOH, and no catalysts. (c) GC-MS analysis of the ^13^CO_2_ source under isotope measurement with ^13^CO and ^13^CH_4_ generation, and ^12^CO and ^12^CH_4_ generation in the insert. (d) Light and ultrasonic power-dependent catalytic gas production of H_2_, CO, and CH_4_ (for 3h). (e) Photocatalytic H_2_, CO, and CH_4_ yield with PET, EG, and TEOA used as electron donors and CO_2_-free input.

We have added the photocatalytic experiment and performance discussion in Figure S17, Supporting Information. Focusing on hydrogen production by comparing photocatalytic H_2_O and H_2_O-CO_2_ reduction conditions, the SNCN/GQD@CdS shows a significant enhancement in only H_2_O substrate without CO_2_ input (Figure S17a, Supporting Information). It suggests competitive photocatalytic kinetics between H_2_O and CO_2_ reduction, bringing about undesirable H_2_ yield in this system.

It also records the photocatalytic performance with different conditions of only TPA, no PET, no NaOH, and no catalysts, monitoring gas production of H_2_, CO, and CH_4_ in Figure S17b, Supporting Information. As the control conditions experiments, compared to the negligible amount of H_2_, CO, and CH_4_ in the air (far less than 1 μmol∙g^-1^), the factors of PET sacrifice agent, alkaline environment, and catalysts are necessary for catalytic PET-enhanced H_2_O and CO_2_ conversion. More specifically, upon the comparison of TPA, EG, and PET applied as catalytic conditions, the gas yield of TPA resembles that of no PET, no NaOH, and no catalysts, indicating TPA cannot be capable of acting as an electron donor.

We have analyzed the sources of H and C in detected H_2_, CO, and CH_4_ by a series of characterization techniques of gas generation contrast and isotope measurement. The source of H mainly results from H_2_O, by comparing the H_2_ generation under the two different sacrificial agents of PET and TEOA (triethanolamine) substrates in photocatalytic behavior in Figure S17e, Supporting Information. A little H_2_ gas also originates from the persistent dehydrogenation behavior of EG monomer signifying the hydrolysis product of PET, which has been in-depth discussed in Figure S16, Supporting Information. The isotope experiments illustrate the gas products of CO and CH_4_ not only from CO_2_ but also from EG with the ^13^CO_2_ source (Figure S17c, Supporting Information). Meanwhile, upon the difference between CO_2_ source input and CO_2_-free input to monitor CO and CH_4_ yields (Figure S17e, Supporting Information), the gas production under PET, EG, and TEOA substrates has a considerable enhancement than that of no CO_2_ condition, turning out gaseous C source mostly from CO_2_. The mechanism of a small amount of gaseous C originating from the oxidation of EG is explained in Figure S16, Supporting Information.

By the comparative experiment of controlling the light intensity and ultrasound power in Figure S17d, Supporting Information, we can find that the catalytic performance of gas production growth is linear of H_2_, CO, and CH_4_ with the enhancement of light and ultrasound field. The catalysts of SNCN/GQD@CdS have the photo/sono synergistic effect, for which photogenerated charge carriers and ultrasonic cavitation effect are responsible. Afterward, the gas production is affected by the purity and types of the sacrificial agent, such as different substrates of PET, TEOA, and EG in Figure S17e, Supporting Information.


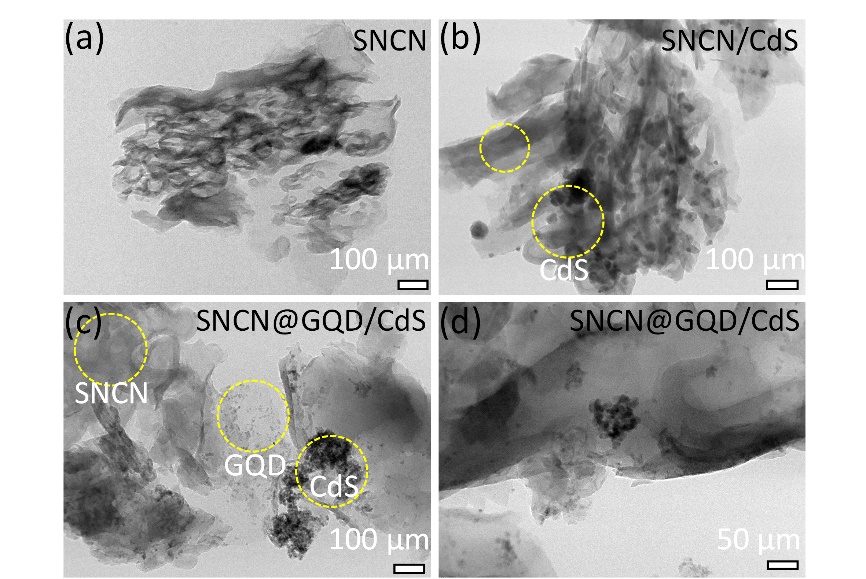


**Figure S18.** TEM images before photocatalysis behavior of SNCN (a), SNCN/CdS (b), SNCN@GQD/CdS (c), and the magnified SNCN@GQD/CdS (d).


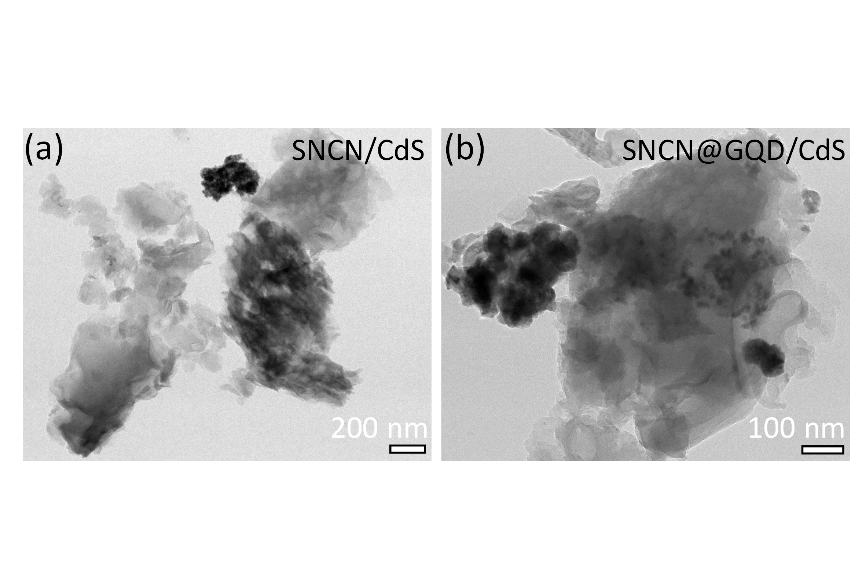


**Figure S19.** TEM images after photocatalysis behavior of SNCN/CdS with photocorrosion of CdS (a) and SNCN@GQD/CdS with stable CdS (b).

The metal-ionic leakage toxicity performance test directly reflects the interfacial interaction bonding between the catalysts and the substrate, as well as the hazard of photo corrosion induced by photo-oxidation of S^2-^. CdS-based catalysts have been facing severe stability issues and the primary hazard is derived from the photo corrosion of CdS considered. S^2-^ is more susceptible to photo-corrosion when it absorbs photons, resulting from oxidation by photo-generated holes. The self-oxidation can bring about high carrier recombination, low photocatalytic activity, and even provoke Cd^2+^ leakage in the aqueous solution.


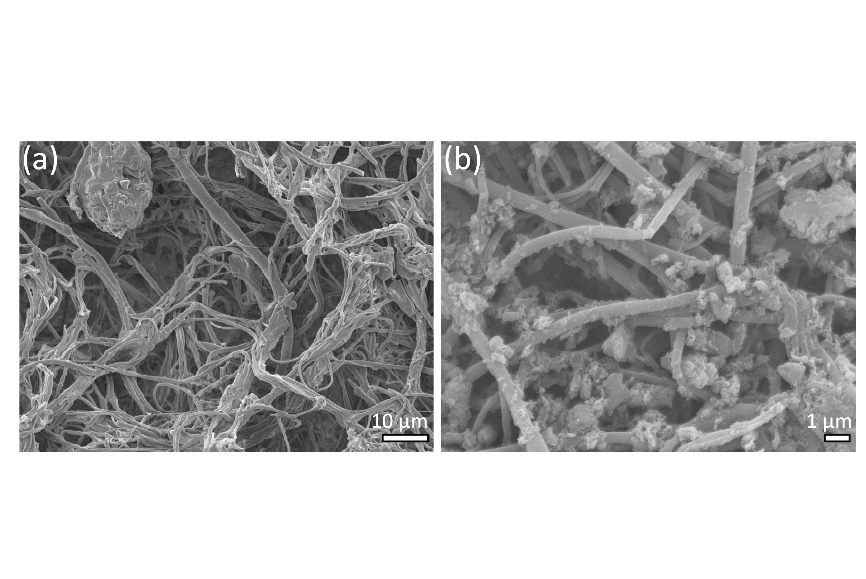


**Figure S20.** SEM photographs of the smooth surface of the nanofibers of SNCN@GQD/CdS/MTSJA nanocomposites before (a) and after (b) photocatalysis behavior.


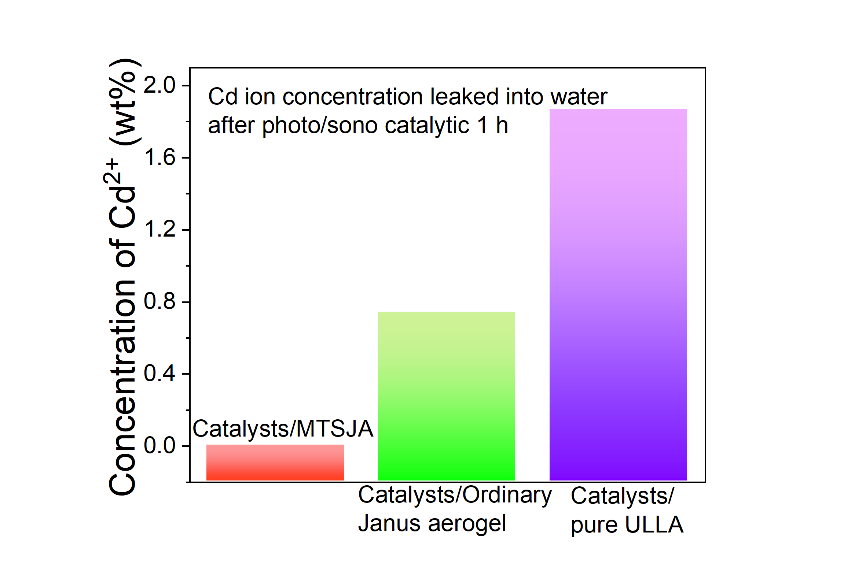


**Figure S21.** Cd ion concentration leakage measurement after photo/sono catalysis by the different aerogels of MTSJA, ordinary Janus aerogel, and pure ULLA to support the SNCN@GQD/CdS catalysts by the inductively coupled plasma technology.

The SNCN/GQD@CdS are loaded on different aerogels of mortise-and-tenon aerogel, ordinary Janus aerogel, and pure hydrophilic aerogel. Undergoing the same catalytic reaction conditions, the minimum Cd^2+^ leakage appears in the catalysts/MTSJA measurement, compared with massive Cd^2+^ leakage on the catalysts/ordinary Janus aerogel and catalysts/pure ULLA.


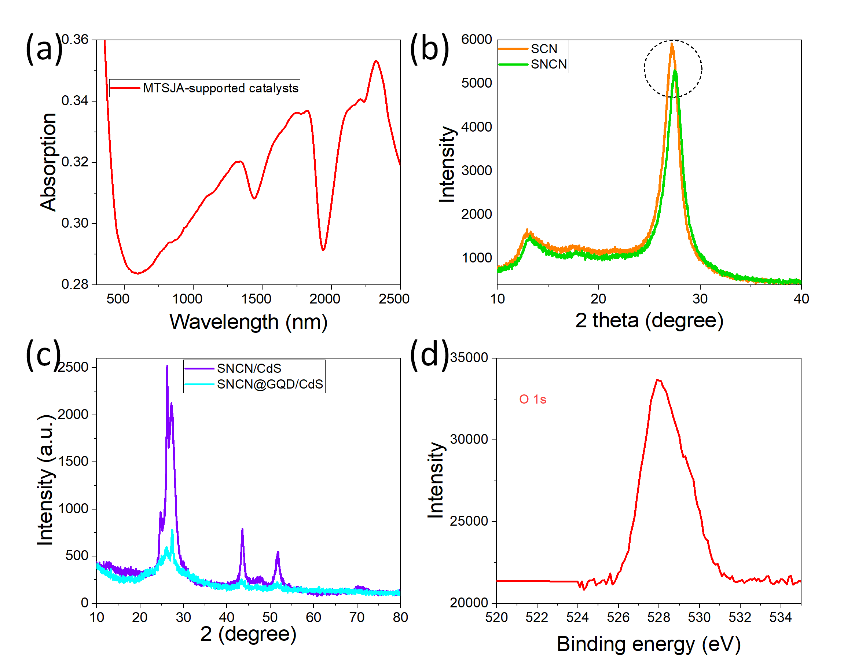


**Figure S22.** (a) The UV-vis-IR spectrum of MTJSA-supported catalysts. (b-c) The amplifying comparison of XRD characterization SCN and SNCN, as well as SNCN/CdS and SNCN@GQD/CdS. (d) The oxygen vacancy of SNCN@GQD/CdS by the XPS characterization.


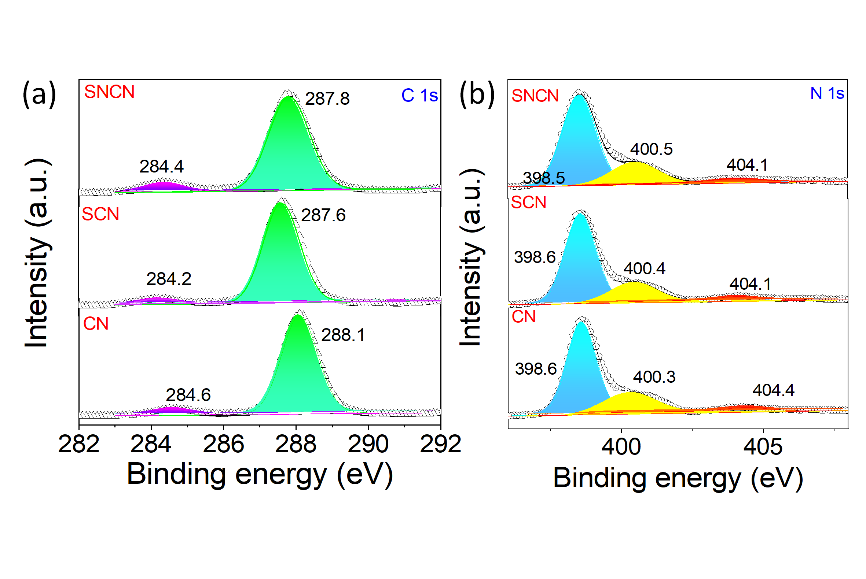


**Figure S23.** High-resolution peak-fitting XPS spectra of C 1s (a) and N 1s (b) of the CN, SCN, and SNCN samples.


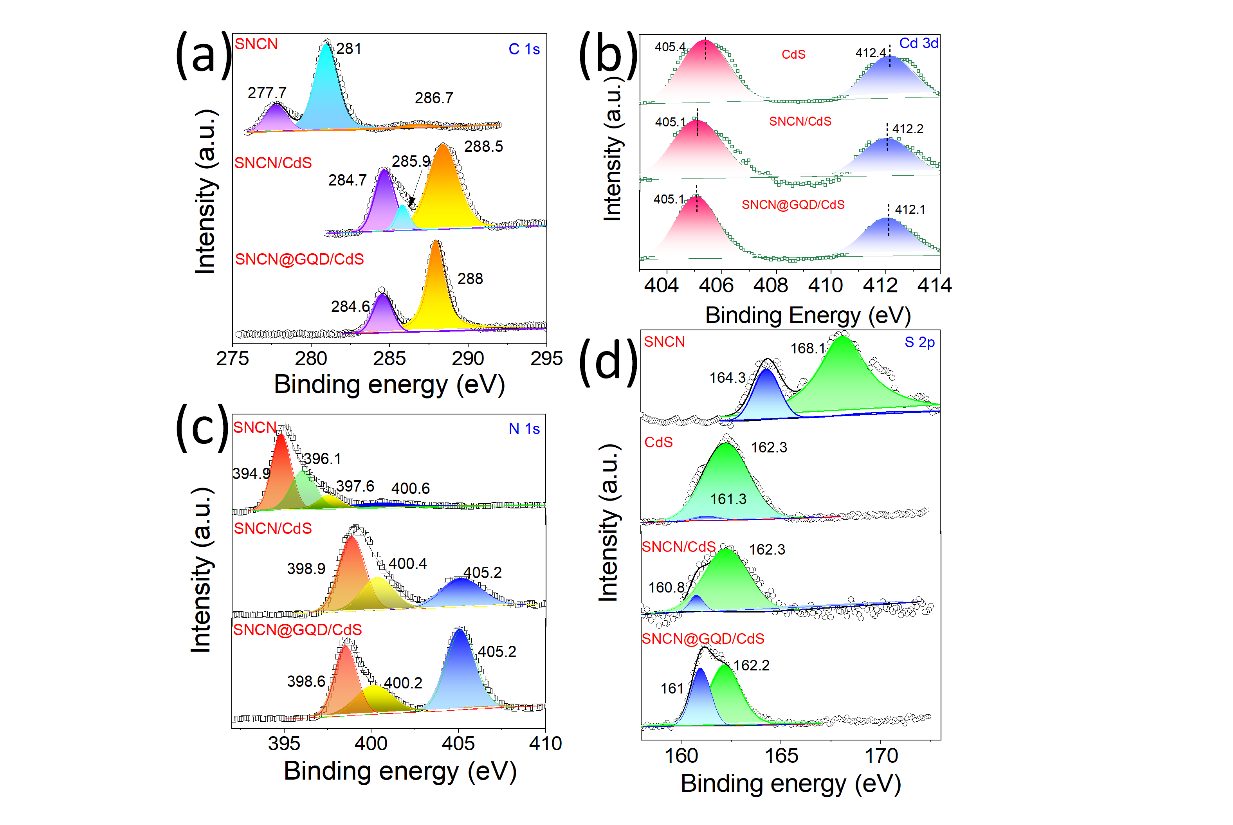


**Figure S24.** High-resolution peak-fitting XPS patterns of SNCN, SNCN/CdS and SNCN@GQD/CdS samples. C 1s (a), Cd 3d (b), N 1s (c) and S 2p (d).


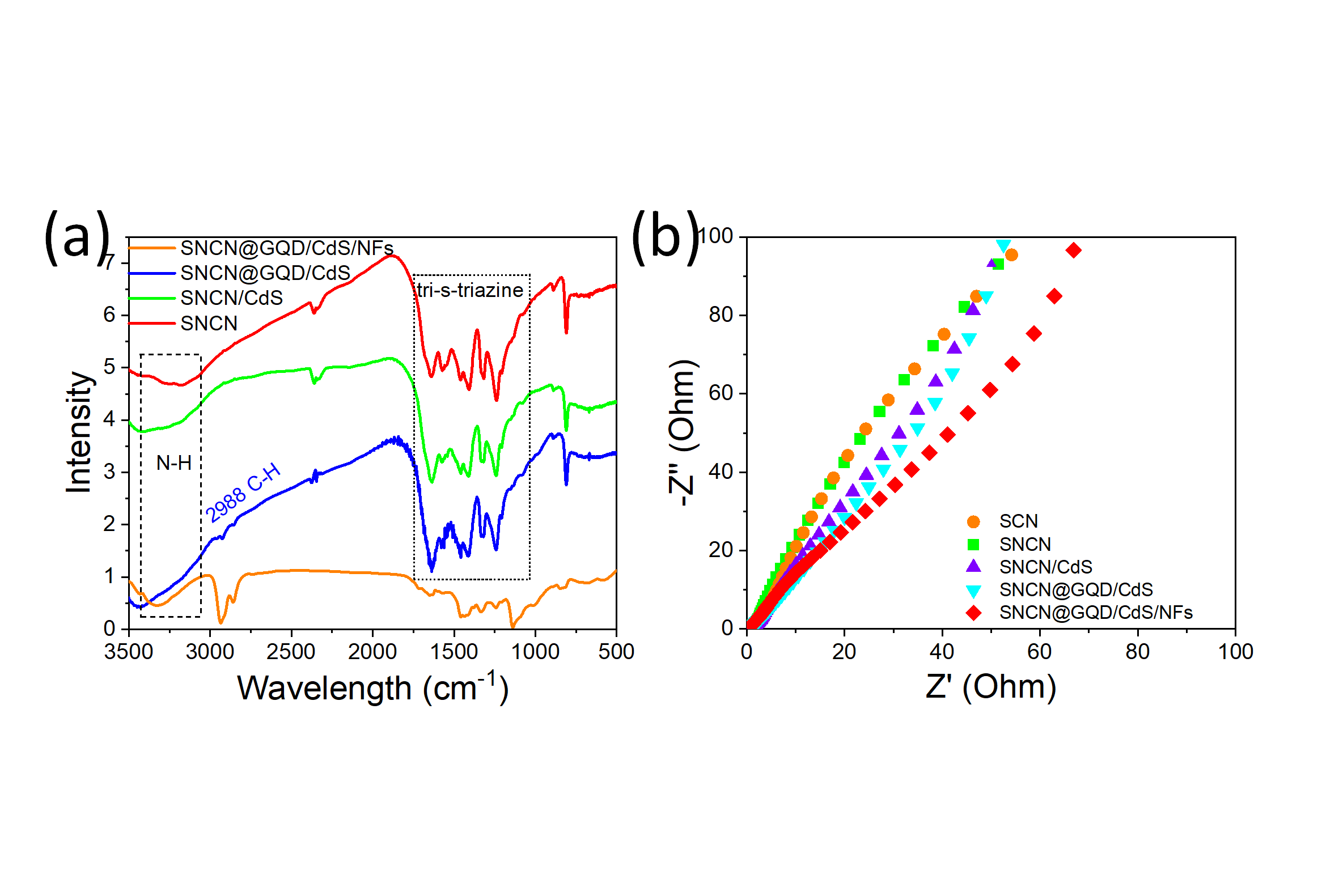


**Figure S25.** FT-IR (a) and electrochemical impedance spectroscopy (b) characterization of different composites of SCN, SNCN, SNCN/CdS, SNCN@GQD/CdS and SNCN@GQD/CdS/NFs.

The FT-IR can analyze surface functional group information, and the SNCN sample has six main characteristic peaks at 830 cm^-1^, 1294 cm^-1^, 1358 cm^-1^, 1441 cm^-1^, 1511 cm^-1^, and 1600 cm^-1^, which are associated with tri-s-triazine structure breathing peaks and skeletal vibration of C-N bond of SNCN. N-H vibration at 3217 cm^-1^ and 3370 cm^-1^ of the SNCN sample appears a decrease with the addition of CdS. The typical characteristic peak at 2988 cm^-1^ belongs to the C-H bending vibration of GQD (Figure S25a, Supporting Information). Besides, the electrochemical impedance spectroscopy (EIS) test accounts for the charge transfer ability corresponding to carrier separation resistance in the semiconductor interface. Usually, Nyquist curves record radius directly proportional to the relative carrier separation resistance (Figure S25b, Supporting Information).


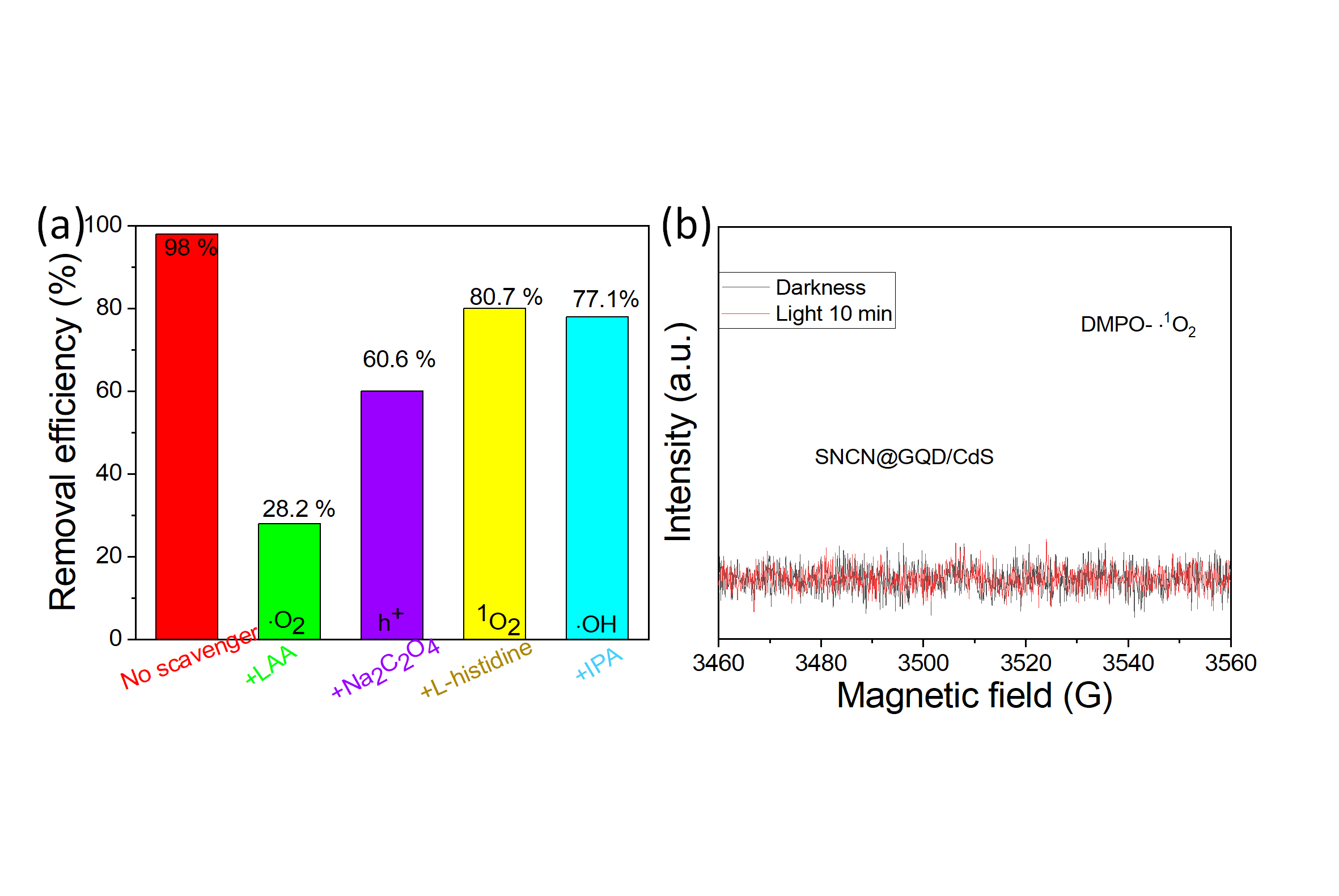


**Figure S26.** (a) Effects of different scavengers on methylene blue degradation efficiency over the catalysts. (b) ESR measurement of DMPO- ∙^1^O_2_.


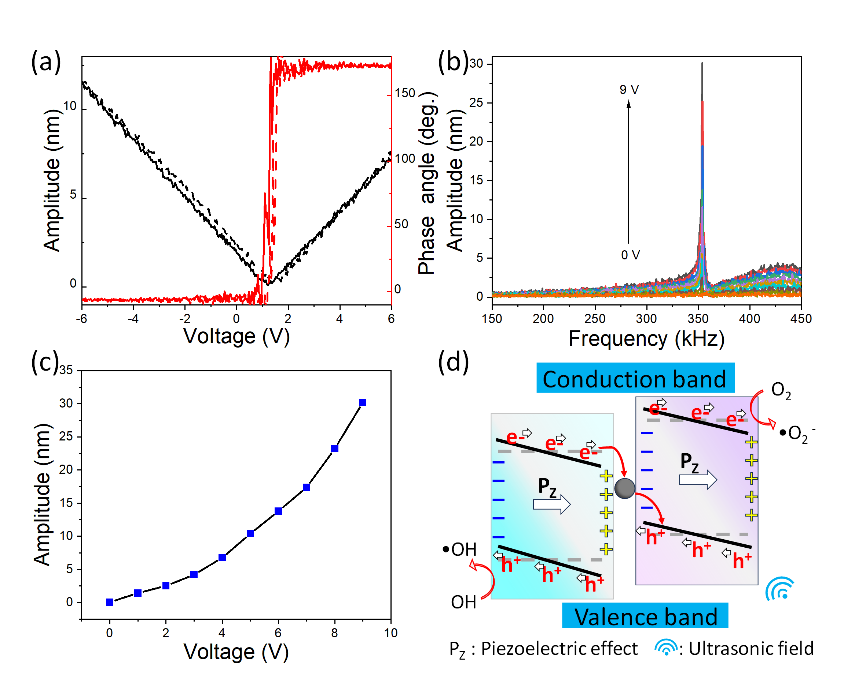


**Figure S27.** Piezoelectric force microscopy measurement of SNCN/GQD@CdS catalysts. (a) A typical butterfly ring of amplitude loop and phase change the response of reversal hysteresis loops to bias voltage field. (b-c) Amplitude intensity response to bias voltage. (e) The mechanism diagram of piezoelectric catalysis.

The mechanism of the piezoelectric effect is revealed. The piezoelectric effects of the Z-type SNCN/GQD@CdS have been proved by piezoelectric force microscopy (PFM) in Figure S27, Supporting Information. A typical butterfly ring of amplitude loop in black line and 169° phase change response of reversal hysteresis loops in red line exhibit under 10 V bias voltage field measurement, which implies the ferroelectric domain characteristic and representative polarization switching activity of the SNCN/GQD@CdS heterojunction (Figure S27a, Supporting Information). The amplitude appears as stimuli response behavior under variational applied bias voltage (Figure S27b, Supporting Information), and the resonant intensity rises as the growth of voltage (Figure S27c, Supporting Information). It demonstrates that the SNCN/GQD@CdS composites possess excellent piezoelectric response capabilities. The piezocatalysis of the Z-type system is primarily governed by the band theory and is determined by two important factors, namely controlling band bending and carrier migration manipulation (Figure S27d, Supporting Information).


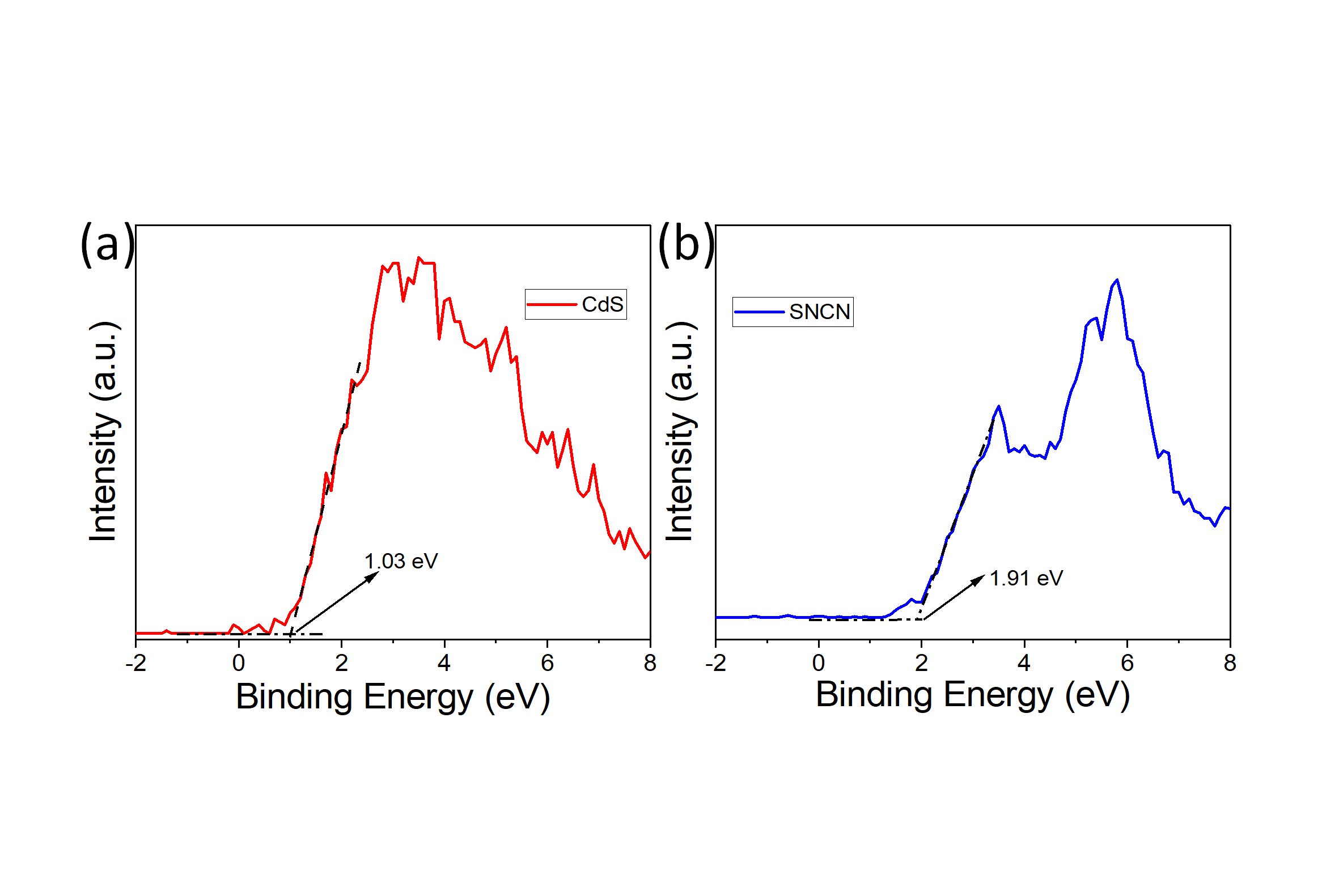


**Figure S28.** XPS patterns of the valence band characterization of CdS and SNCN samples.


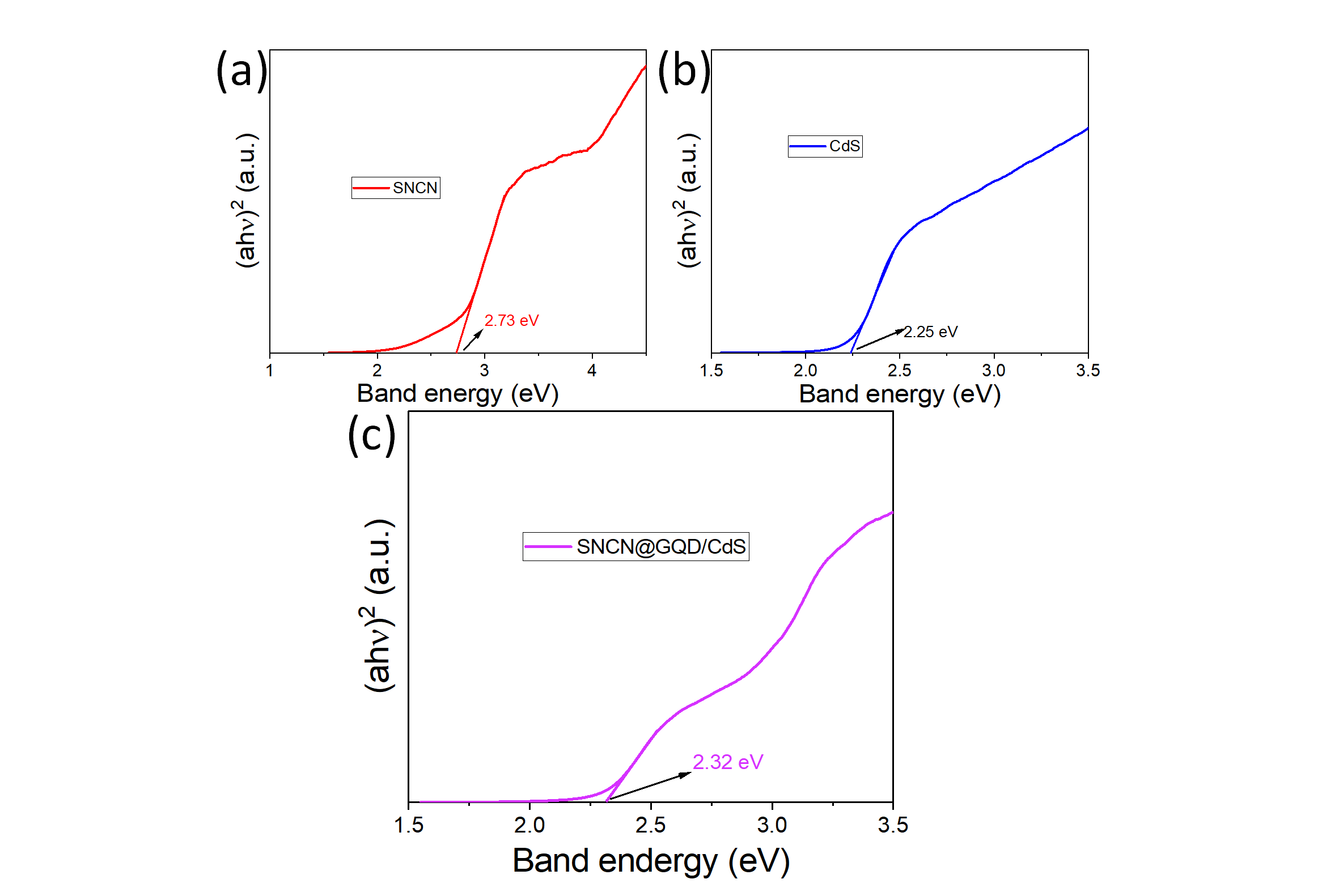


**Figure S29.** The plots of (*αhν*)^1/2^ versus *hν* of SNCN, CdS and SNCN@GQD/CdS samples.

**Reference**

[1] M. Zhu, Y. Li, G. Chen, F. Jiang, Z. Yang, X. Luo, Y. Wang, S. D. Lacey, J. Dai, C. Wang, C. Jia, J. Wan, Y. Yao, A. Gong, B. Yang, Z. Yu, S. Das, L. Hu, *Adv. Mater.* **2017**, 29, 1704107;

[2] Y. Yang, H. Zhao, Z. Yin, J. Zhao, X. Yin, N. Li, D. Yin, Y. Li, B. Lei, Y. Du, W. Que, *Materials Horizons* **2018**, 5, 1143;

[3] L. Shi, W. Tao, N. Zheng, T. Zhou, Z. Sun, *Applied Thermal Engineering* **2023**, 230, 120770;

[4] N. Xu, X. Hu, W. Xu, X. Li, L. Zhou, S. Zhu, J. Zhu, *Adv. Mater.* **2017**, 29, 1606762;

[5] J. Zhao, Y. Yang, C. Yang, Y. Tian, Y. Han, J. Liu, X. Yin, W. Que, *Journal of Materials Chemistry A* **2018**, 6, 16196;

[6] Z. Liu, H. Song, D. Ji, C. Li, A. Cheney, Y. Liu, N. Zhang, X. Zeng, B. Chen, J. Gao, Y. Li, X. Liu, D. Aga, S. Jiang, Z. Yu, Q. Gan, *Global Challenges* **2017**, 1, 1600003;

[7] Q. Xia, C. Wang, N. Xu, J. Yang, G. Gao, J. Ding, *Adv. Funct. Mater.* **2023**, 33, 2214769;

[8] L. Ying, F. Liang, Z. Huang, J. Ding, W. Wang, S. Liu, J. Lu, *Chem. Eng. J.* **2023**, 474, 145709;

[9] K. Sheng, M. Tian, J. Zhu, Y. Zhang, B. Van der Bruggen, *ACS Nano* **2023**, 17, 15482;

[10] Y. Guo, H. Lu, F. Zhao, X. Zhou, W. Shi, G. Yu, *Adv. Mater.* **2020**, 32, 1907061.
